# Pesticide residues in daily bee pollen samples (April–July) from an intensive agricultural region in Southern Germany

**DOI:** 10.1007/s11356-020-12318-2

**Published:** 2021-01-11

**Authors:** Carolin Friedle, Klaus Wallner, Peter Rosenkranz, Dieter Martens, Walter Vetter

**Affiliations:** 1 Apicultural State Institute, University of Hohenheim, Stuttgart, Germany; 2 Agricultural Research and Development Institute, Speyer, Germany; 3 Institute of Food Chemistry (170b), University of Hohenheim, Stuttgart, Germany

**Keywords:** Bee pollen, Pesticide residues, Pollen hazard quotient, QuEChERS, LC-MS/MS, Germany

## Abstract

Insect-pollinated plants are essential for honey bees to feed their brood. In agricultural landscapes, honey bees and other pollinators are often exposed to pesticides used for cultivation. In order to gain more insight into the fluctuation of pesticide loads, 102 daily pollen samples were collected between April and July 2018 in a fruit-growing area in Southern Germany. Samples were analyzed with respect to more than 260 pesticides using a multi-residue pesticide analysis method. Almost 90% of the analyzed pollen samples featured between one and thirteen different pesticides. In total, 29 pesticides were detected at maximum concentrations of up to 4500 ng/g pollen. Maximum residual concentrations of most pesticides were observed during April and the first half of May, as well as during the second half of June. In most cases, serial data of pesticide residuals were detected for approximately 10 subsequent days with two or three maximum values, which were several folds higher than concentrations on the days before and thereafter. The pollen hazard quotient (PHQ) was calculated to estimate the risk of the detected pesticides to honey bees and wild pollinators.

**Supplementary Information:**

The online version contains supplementary material available at 10.1007/s11356-020-12318-2.

## Introduction

Apart from nectar, pollen from plants is essential to honey bees (*Apis mellifera)* for feeding their brood. From early spring on, bees start collecting pollen from blooming crops, e.g., willows, fruits, vegetables, and flowers, and transporting it in the pollen baskets known as corbicula via their legs to the hive (Kevan and Baker 1983; Willmer 2011). After the addition of nectar and bee secretion, pollen is stored in comb cells (Nagai et al. 2005; DeGrandi-Hoffman et al. 2013). The stored pollen, called bee bread, can be stored over months, but bees prefer to consume freshly stored pollen within 2 to 4 days (Anderson et al. 2014; Carroll et al. 2017). Bee bread is then used by nurse bees to produce larval food (Lindauer 1952; Cridge et al. 2015). However, bee bread and bee pollen were shown to be frequently contaminated by pesticides (Lindauer 1952; Chauzat et al. 2006; Botías et al. 2015; Traynor et al. 2016; Codling et al. 2018; Böhme et al. 2018, 2019; German Bee Monitoring 2014–2019). This is due to the fact that honey bees are collecting nectar and pollen not only from wild plants but mainly from crops or plants used in agricultural industry (McGregor 1976).

Residues of agricultural pesticides in pollen can originate from the application of systemic compounds before the blooming period, from contamination of water and soil as well as from spray application to the blooming plants (Aktar et al. 2009; The Scottish Government 2018). To protect orchards and oilseed against pests and fungal growth, spray applications of “pesticide cocktails” are recommended in Germany with spraying regimes of up to 15 different applications from early spring to late summer (Wallner 2012; Roßberg and Harzer 2015). The contamination status of bee pollen can be monitored by means of a pollen trap installed at the front of a hive entrance which samples up to 40% of the daily amount of pollen brought to the hive by foraging bees (Keller et al. 2005). Most studies to date have been performed on pooled samples, typically collected from weekly trappings, at single timepoint from multiple colonies in the same apiary, or multiple collections are pooled over an entire season (Drummond et al. 2018; Tosi et al. 2018). Little is known about the pesticide load in daily samples of bee pollen. Böhme et al. (2018) analyzed in between 9 and 39 of daily bee pollen samples collected from March to August over a time course of 5 years, at three different agricultural sites (“meadow” with about 60% permanent grassland, “grain” with high percentages of grains, and “fruit” with 30% permanent crops) in Southern Germany. Random analysis of at least one sample each week indicated highest pesticide concentrations at the “fruit” site with 7200 ng/g pollen. These concentrations were much lower than those reported in two studies from the USA where pesticide concentrations in weekly pollen samples mounted up to 99,000 ng/g pollen (Mullin et al. 2010; Stoner and Eitzer 2013). Likewise, the pesticide load in bee pollen could be traced back to pesticide spraying in different cultivation areas. Nevertheless, detailed knowledge about the development of pesticide concentrations in pollen samples during a spring collecting season is still lacking. Furthermore, frequency and time course of remaining pollen contamination was vastly unknown for the time period after occurrence of the maximum contamination level.

The goal of this project was to study the distribution and progression of pesticide contamination in daily bee pollen samples throughout an entire growing season (April to July 2018) at a representative bee hive located within a fruit cultivation area in Southern Germany. For this purpose, daily samples were collected from a single hive by means of a pollen trap from April to July 2018. Samples were analyzed for over 260 pesticides by LC-MS/MS, including almost all as generally used in Germany. The data collected was used to elucidate number of different pesticides, their frequency, and maximum concentrations in pollen, as well as record their reoccurrence during the entire growing season to understand pesticide fluctuations in an agricultural landscape.

## Materials and methods

### Site description and collection

**Fig. 1:**
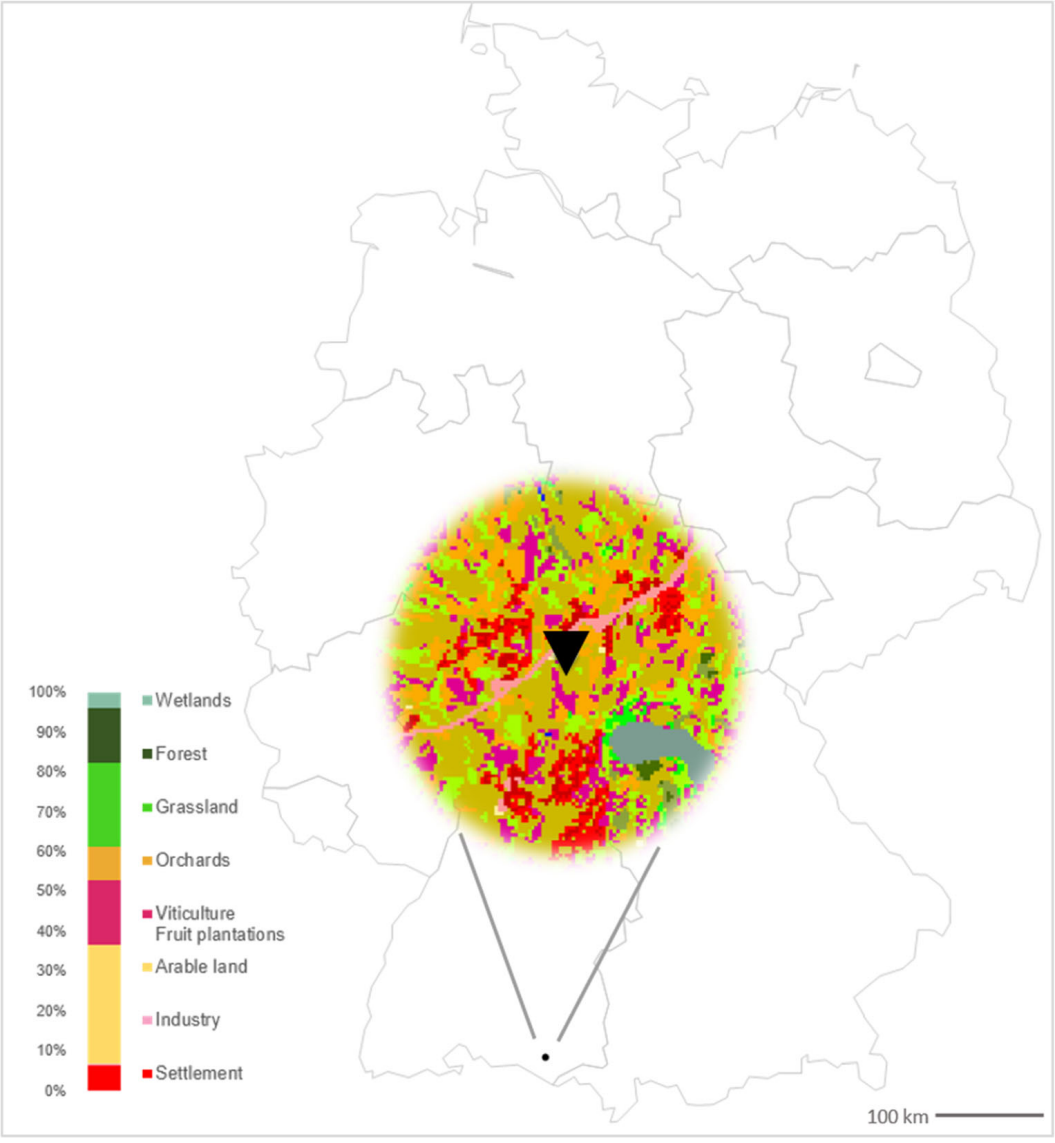
Location area of sampling (*r* = foraging distance 1500 m) in Southern Germany (mapping with JMP® pro 15.0; landscape use: basis data from the Environmental Information System (UIS) of the State Institute for the Environment Baden-Wuerttemberg; evaluation with GIS, geonline GmbH)

One individual bee colony was used for sampling. Since a colony usually consists of more than 30,000 bees, typically ~ 20% of the workers are engaged in foraging pollen on a given day (Klein et al. 2019). This scenario seemed to be appropriate in order to get first impressions about the amount of pesticides honey bees become daily exposed to over an entire season. An apiary on personal grounds was selected for this study (exact coordinates of the apiary will not be shown and no permits were needed for this study); it is located in an intensive fruit cultivation area nearby Friedrichshafen (Baden-Wuerttemberg, Southern Germany) (Fig. 1). The area around the hive within the mean foraging distance of foraging bees of around 1500 m (Steffan-Dewenter and Kuhn 2003) is characterized by a low population density (population 325/km^2^) (Statistical Service Office Baden-Wuerttemberg 2020) and cultivation of different crops. Apples, sweet and sour cherries, and plums with over 0.25 km^2^ agricultural area (Info Service of Agricultural - Nutrition and Rural Areas 2018; Statistical Service Office Baden-Wuerttemberg 2017) are cultivated in this area. Bee pollen traps were installed to collect pollen loads from returning honey bees (*Apis mellifera*) (Detroy and Harp 1976) (Figure S1). From April (starts at April 12 with numeric D1) until July 31 (D102), 2018, daily pollen samples were collected on 102 out of 111 consecutive days (except nine rainy days without foraging activities) by a volunteer beekeeper. Daily bee pollen samples (20 to 55 g) were homogenized, and aliquots of 20 g were removed and stored at − 20 °C in polyethene sample bags until sample preparation.

### Chemicals

Acetonitrile (for LC-MS, ≥ 99.95%), nonane (for synthesis, ≥ 99%), and fine magnesium sulfate (≥ 99%) were from Carl Roth (Karlsruhe, Germany). The sorbent Sepra C_18_-E (50 μm, 65 Å) and Sepra PSA (51 μm, 73 Å); the “roQ QuEChERS Kit” with 4.0 g magnesium sulfate, 1.0 g sodium chloride, 1.0 g sodium citrate tribasic dihydrate, and 0.5 g sodium citrate dibasic sesquihydrate; and Sepra graphitized carbon black (laboratory use) were from Phenomenex (Karlsruhe, Germany). The internal standard solution was prepared in-house at the Agricultural Research Development Institute (LUFA Speyer Germany) with triphenyl phosphate and *d*_*10*_-chlorpyrifos (20 ng/μL acetonitrile each) (HPC, Bohnsdorf, Germany). Also, the pesticide standard solution with all 262 analytes (HPC, Bohnsdorf, Germany, and Sigma-Aldrich, Darmstadt, Germany) at concentration 1 ng/μL acetonitrile (Table S1) was prepared at the Agricultural Research Development Institute (LUFA, Speyer).

### Sample preparation for pesticides analysis

Pollen samples were brought up to room temperature, mechanically homogenized in a mortar, and subsequently dried for 8 h at 30 °C in a heating cabinet (Binder, Tuttlingen, Germany). An aliquot of 5 g ± 0.001 g pollen sample was weighed into a 50-mL tube (Buddeberg, Mannheim, Germany), and the following QuEChERS method (Anastassiades et al. 2003) (§64 LFGB, BVL L 00.00-115/1:2015-03) with individual modifications was used. Ten milliliter demineralized water, 10 mL acetonitrile, and 10 μL internal standard solution were added, and the mixture was mechanically shaken for 20 min at 400 rpm on a Heidolph Instruments Promax 2020 (Schwabach, Germany). After centrifugation (10 min at 2750 x G), the supernatant was transferred into a new 50-mL tube, which contained 4.0 g magnesium sulfate, 1.0 g sodium chloride, 1.0 g sodium citrate tribasic dihydrate, and 0.5 g sodium citrate dibasic sesquihydrate (roQ QuEChERS Kit). The sample solution was shaken for 2 min followed by centrifugation for 10 min at 2750 x G. The supernatant was placed in a 15-mL tube (Buddeberg, Mannheim, Germany) which already contained 0.5 g fine MgSO_4_. The sample was shaken for 1 min and centrifuged (10 min at 2750 x G). Then, 5 mL of the supernatant was transferred into a glass tube and supplemented with 50 μL nonane solution (10 g nonane/50 mL acetone). Afterwards, the sample solution was evaporated to near dryness in a heating block maintained at 40 °C under nitrogen flow. The residue was resuspended in 2.5 mL acetonitrile and swiveled for 30 s in an ultrasonic bath. The solution was then transferred into a new 15-mL tube containing 0.49 g cleaning mixture (0.31 g magnesium sulfate, 0.06 g Sepra C_18_-E, 0.11 g Sepra PSA, and 0.01 g graphitized carbon black). The sample was shaken for 1 min and centrifuged for 10 min at 2750 x G. A 1 mL aliquot from the supernatant was transferred into a 1.5 mL LC vial (Macherey-Nagel, Düren, Germany) and stored at − 20 °C until analysis.

### Palynological analysis

A palynological analysis of four selected samples D9-D13 (April 22–26) was prepared as follows. An aliquot of 100 mg homogenized pollen was weighed into a 50-mL tube containing 10 mL demineralized water and a drop of dish soap. The mixture was shaken for 1 min, and a drop was transferred to an object carrier, dried, and covered with Kaiser’s glycerol gelatin for microscopy (Merck, Darmstadt, Germany).

### High performance liquid chromatography with tandem mass spectrometry (LC-MS/MS) analysis of pesticides

LC-MS/MS analyses were performed with an API 400 system (AB Sciex, Darmstadt, Germany) at Agricultural Research and Development Institute (LUFA, Speyer, Germany). Analytes were separated on a Gemini NX C_18_ column (100 mm length × 3 mm inner diameter, 3 μm particle size; Phenomenex, Karlsruhe, Germany) at 30 °C. Each 10 μL of sample was injected to the column equilibrated with 5 mmol/L ammonium acetate and 0.1% formic acid in water (A) at a flow rate of 300 μL/min. A linear gradient, within 3 min, of 30 to 70% (B) (methanol, 5 mmol/L ammonium acetate) in A was run, followed by an increase to 100% (B) within 10 min. After 2 min at 100% (B), the ratio was turned back to 70% and held for another 5 min. MS/MS measurements were performed in multiple reaction monitoring (MRM) mode using electrospray ionization (ESI) in the positive mode with an ion spray voltage of 5500 V and a desolvation temperature of 400 °C. A total of 262 analytes (pesticides and related compounds) were subjected to this analytical method (Table S1).

### Quality control

One sample fortified with analytes was run per batch of 25 samples in a following manner: blank pollen (5 g pollen sample without detectable residues, as checked previously) was mixed with 10 mL demineralized water and 9.75 mL acetonitrile, spiked with 250 μL pesticide standard solution and 10 μL internal standard solution (absolute concentration 200 ng triphenyl phosphate and *d*_*10*_-chlorpyrifos each), and prepared as explained above in “Sample preparation for pesticides analysis”. All samples were prepared within 1 week, one batch (25 samples + 1 blank pollen) per day. The recovery was calculated using a defined final volume of 2.5 mL (after evaporation) by comparison with the absolute internal standard. Recovery rates were calculated for four quality control samples in total, revealing a range between 29 and 160% per substance (except spirodiclofen, exhibiting a significant low recovery rate of 4%). The mean standard deviation between replicates was 14% (Table S2).

### Toxicological evaluation

In order to assess the hazard to honey bees of pesticide residues in the pollen samples, the pollen hazard quotient (PHQ) was used following the method of Stoner and Eitzer (2013). The PHQmax is calculated by dividing the maximum concentration (ng/g) of each pesticide detected in the samples by the known LD_50_ value (honey bee oral; μg/bee) as listed in the University of Hertfordshire pesticides properties database (Pesticide Properties DataBase - PPDB 2020). The total PHQ per day (tPHQday) was calculated as the sum of all PHQs of pesticides in the representative day sample. Based on a daily consumption of up to 9.5 mg bee bread by a nurse bee (Rortais et al. 2005), a PHQ of ≥ 50 was considered “relevant” for bee health according to Böhme et al. (2018). With a PHQ of 100, 0.1% of the LD_50_ would be ingested in 1 day, or 1% of the LD_50_ in a 10-day nursing period (Stoner and Eitzer 2013).

## Results

### General observations

Between April and July 2018, 102 daily pollen samples were collected and analyzed for 262 active substances (75 fungicides, 95 herbicides, 89 insecticides, and 3 plant regulators). Altogether 29 pesticides were detected, 15 fungicides, 12 insecticides, and 2 herbicides (Table 1), while the other 258 compounds for which we screened were not detected in any sample. Only 13 daily pollen samples contained no detectable pesticide residues, whereas 89 pollen samples contained between one and thirteen residues per sample (Table S3). The median number of detected residues was five per daily sample. More detected residues, between seven and thirteen per sample, were found from D8 to D17 (April 21–30). Of the 29 individual pesticides detected, these were found in daily pollen samples anywhere on 3 to 64 occasions. The median frequency of detection was 14. The greatest frequency of detection of 64 (*d*_*f*_ in days) was observed for the fungicide trifloxystrobin (Figure S2). The maximum concentrations of the 29 positive detected pesticides ranged between 6 and 4530 ng/g pollen, while tebuconazole showed the greatest concentration of all pesticides with 4530 ng/g pollen in one sample (Table 1). Furthermore, the highest total pesticide concentrations per day sample, between 3300 and 8800 ng/g pollen, occurred in samples between D10 and D12 (Table S3). The day-to-day progression of individual pesticide concentrations in pollen samples is discussed in the following subchapters. Pesticides were divided into their classes of fungicides, herbicides, and insecticides.

**Table 1:** Summary of 29 pesticides detected in 102 daily bee pollen samples during April–July 2018 in Southern Germany

Substances	Class^a^	Group	Application area in Germany^b^	LOQ (ng/g)	Recovery rate (%)	Frequency of detection (*d*_*f*_)	Max. detected concentration (ng/g)	Mean detected concentration (ng/g)	LD_50_ (μg/bee)^c^	PHQmax	Mean PHQ
Acetamiprid	I	Neonicotinoids	f; v	3	84	11	31	1.3	15	2.1	0.09
Azoxystrobin	F	Quinone outside inhibitors	f; v	3	123	6	14	0.6	25	0.6	0.03
Boscalid	F	Succinate dehydrogenase inhibitors	f; v; r	3	105	36	201	7.9	166	1.2	0.05
Chlorantraniliprole	I	Diamides	f; v	3	120	25	307	13.6	104	3.0	0.13
Cyprodinil	F	Anilino-pyrimidines	f	3	29	9	193	5.8	113	1.7	0.05
Difenoconazole	F	Demethylation inhibitors	f; v	3	87	10	48	1.5	177	0.27	0.01
Diflubenzuron*	I	Chitin synthesis inhibitors	f; v; c (B1)	3	79	2	121	1.3	9.1	13	0.14
Dimethenamid	H	Chloroacetamides	f; v; c	3	98	11	121	2.5	118	1.0	0.02
Dimoxystrobin	F	Quinone outside inhibitors	r	3	108	2	6.1	0.1	79	0.08	0.001
Fenhexamid	F	Sterol biosynthesis inhibitors	f; v	10	44	10	274	6.3	102	2.7	0.06
Fenoxycarb*	I	Insect growth regulator	f; c (B1)	3	96	14	367	5.0	204	1.8	0.02
Fenpyroximate	I	Pyrazole	f; v; w	3	82	15	99	2.6	119	0.8	0.02
Flonicamid	I	Flonicamid	f; v; r	3	83	19	35	2.4	100	0.4	0.02
Fluopyram	F	Succinate dehydrogenase inhibitors	f; v; w	5	125	27	4050	123	102	40	1.2
Kresoxim-methyl	F	Quinone outside inhibitors	w	5	116	3	10	0.3	110	0.09	0.002
Methiocarb	I	Carbamates	s.t.	3	94	16	14	1.0	0.08	170	12.2
Myclobutanil	F	Demethylation inhibitors	f; v; w	5	106	39	334	11.9	34	9.8	0.4
Penconazole	F	Demethylation inhibitors	f; v; w	3	55	14	24	1.2	112	0.2	0.01
Pendimethalin	H	Dinitroanilines	c	5	53	17	1810	36.6	101	18	0.4
Picaridin	I	Piperidines	i.r.	15	82	15	117	5.8	–	–	–
Pirimicarb	I	Carbamates	f; v; c	3	53	3	25	0.5	4.0	6.3	0.14
Pyraclostrobin	F	Quinone outside inhibitors	f; v; c	5	100	6	49	1.1	110	0.44	0.01
Pyrimethanil	F	Anilino-pyrimidines	f; w; c	5	57	9	52	1.8	100	0.52	0.02
Spirodiclofen	I	Biosynthesis inhibitors	f; v; c (B1)	5	4	29	402	15.8	196	2.1	0.08
Tebuconazole	F	Demethylation inhibitors	f; v; w	5	90	17	4530	98.4	83	55	1.8
Tebufenozide	I	Diacylhydrazines	f; v; w	3	107	18	412	8.7	100	4.1	0.09
Thiacloprid	I	Neonicotinoids	f; v; c	3	77	47	258	14.0	17	15	0.8
Thiophanate-methyl	F	b-Tubulin inhibitors	f; c	10	101	7	59	2.1	115	0.51	0.02
Trifloxystrobin	F	Quinone outside inhibitors	f; v; c	3	112	64	707	40.6	110	6.4	0.37

*, not approved for application in Germany; −, not available

^a^*F* fungicide, *H* herbicide, *I* insecticide

^b^*c* crop seed, *f* fruit cultivation, *i.r.* insect repellent, *r* rapeseed, *s.t.* seed treatment, *v* vegetable cultivation, *w* wine, *B1* is classified as hazardous to bees (Federal Office of Consumer Protection and Food Safety 2020)

^c^LD_50_ honey bees oral (*Apis* sp.), values rounded to two significant numbers (Pesticide Properties DataBase - PPDB 2010)

### Fungicides

A total of 15 fungicides were detected in all pollen samples. The two highest concentrations measured in this study showed tebuconazole and fluopyram with concentrations above 4000 ng/g pollen.

**Fig. 2:**
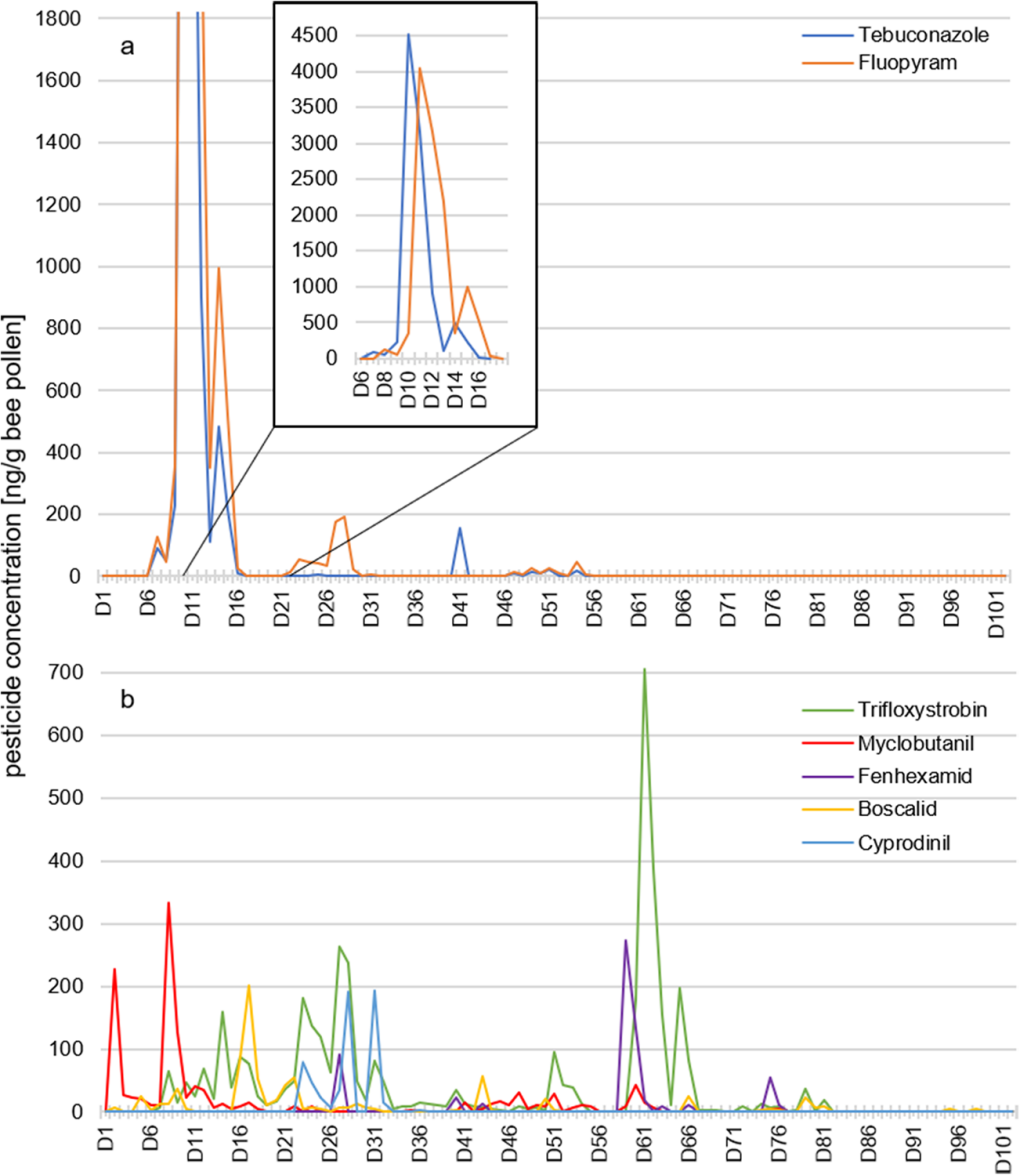
Line chart with a day-to-day progression of fungicides with maximum concentrations (**a**) > 4000 ng/g bee pollen and (**b**) between 100 and 700 ng/g bee pollen

*Tebuconazole (d*_*f*_ *17)*. The greatest single day concentration of 4530 ng/g pollen was observed on D10 (April 23, 2018) (Fig. 2a). This triazole fungicide, normally used against various foliar diseases such as powdery mildew and black spot in fruit and vegetable cultivation and in crop seed (Federal Office of Consumer Protection and Food Safety 2020) (Table 1), was detected in 17 daily pollen samples. The highest values were observed on 10 subsequent days between D7 and D16. Initially detected on moderate concentrations between D7 and D8, tebuconazole concentrations slightly increased on D9 to 230 ng/g pollen. On the following day, a maximum concentration of 4530 ng/g was measured. After 2 consecutive days at a high level, tebuconazole concentrations fell sharply until it was no longer detectable on D17. At the end of May (D41), tebuconazole was detected on a second occasion at 160 ng/g pollen in just one daily pollen sample.

*Fluopyram (d*_*f*_ *27)* is a succinate dehydrogenase inhibitor used in fruit and vegetable cultivation and viticulture (Table 1) and was detected in 27 daily pollen samples. It is noted that the greatest concentration of 4050 ng/g pollen was recorded on the same day as the maximum observed with tebuconazole (D10). This observation and the very similar progress over the same period (Fig. 2a, Figure S3) suggested that both fungicides were possibly applied together. Specifically of note in this context, there is at least one formulation used in Germany for fruit cultivation (Federal Office of Consumer Protection and Food Safety 2020) containing fluopyram and tebuconazole, both with concentrations of 200 g/L. A palynological sample analysis of pollen for days with highest load of fluopyram and tebuconazole (D10 and D11) confirmed high proportions of *Prunus* sp. type (± 77% stone fruit) and *Pyrus* sp. type (± 18% pome fruit) along with scattered contributions of *Acer* sp. (maple) and *Picea* sp. (spruce) pollen (Table S4). Hence, there exists strong evidence that both pesticides were also distributed and collected at similar ratio during fruit cultivation. In the beginning of May, fluopyram was detected again on 10 subsequent days (D22 to D31). However, the maximum concentration of 190 ng/g pollen was much lower (Fig. 2a), suggesting either the spraying of a lower amount of fluopyram or a lower share of pollen from the treated field in the daily collection of the hive. The absence of tebuconazole in these samples pointed towards the use of a different pesticide formulation. Within this period, the highest concentration was reached on the seventh day.

*Trifloxystrobin (d*_*f*_ *64)* is mainly used in fruit and vegetable cultivation and crop seed (Table 1) and was found most frequently of all pesticides, showing a frequency of detection of 64 out of 102 days with a maximum concentration of 710 ng/g pollen. It was detected consistently between D7 and D49 with only one exception. Within this period, the concentrations increased and dropped again (concentration up to 260 ng/g pollen). Between D22 and D29, trifloxystrobin showed similar concentrations as fluopyram (in its second period). While the resulting constant ration of both fungicides could be accidental, it could also mean that they were applied together. Formulations containing fluopyram together with trifloxystrobin are used in fruit cultivation in Germany (both with concentrations of 250 g/L (Federal Office of Consumer Protection and Food Safety 2020)). The long period and the differing composition provide evidence for application of this fungicide on several days at different locations. In addition, a second period with higher trifloxystrobin levels was observed between D60 and D70, with maximum concentration of 710 ng/g pollen in one sample. It required 9 subsequent days for the concentration to fall below LOQ (Fig. 2b).

*Myclobutanil (d*_*f*_ *39)* is frequently applied as a demethylation inhibitor in fruit and vegetable cultivation and viticulture. It was first observed in this investigation in the samples between D2 and D18. Within these 17 days, the concentrations were usually low, except on D2 (230 ng/g pollen) and D8 (maximum level of 330 ng/g pollen) (Fig. 2b). Again, subsequent detections suggested repeated applications either on the same field or on other local areas. *Fenhexamid (d*_*f*_ *10)*, which also had a low recovery of 44%, was detected between May and July at moderate and lower concentrations. The highest concentrations were measured between D59 and D66, including 4 days showing up to 270 ng/g pollen. Detection of *boscalid (d*_*f*_ *36)* started at a low level in April with up to 200 ng/g pollen on D17, followed by a concentration drop. *Cyprodinil (d*_*f*_ *9)* displayed a low recovery rate of only 29%. Nevertheless, this aminopyrimidine fungicide peaked at 190 ng/g pollen on D28 and was detected at moderate concentrations between D23 and D27 (Fig. 2b).

Further eight fungicides with concentrations < 100 ng/g pollen could be detected in the samples. The majority of these substances (*thiophanate-methyl (d*_*f*_ *7)*, *difenoconazole (d*_*f*_ *10)*, *pyraclostrobin (d*_*f*_ *6)*, *pyrimethanil (d*_*f*_ *9)*, *azoxystrobin (d*_*f*_ *6)*, *kresoxim-methyl (d*_*f*_ *3)*, and *dimoxystrobin (d*_*f*_ *2)*) showed highest concentrations between D2 and D28. *Penconazole (d*_*f*_ *14)* was detected between D58 and D62 and again between D75 and D78 at lower concentrations (Figure S4a).

### Herbicides

Only the following two herbicides were detected with maximum concentrations between 120 and 1810 ng/g pollen in the analyzed pollen samples.

**Fig. 3:**
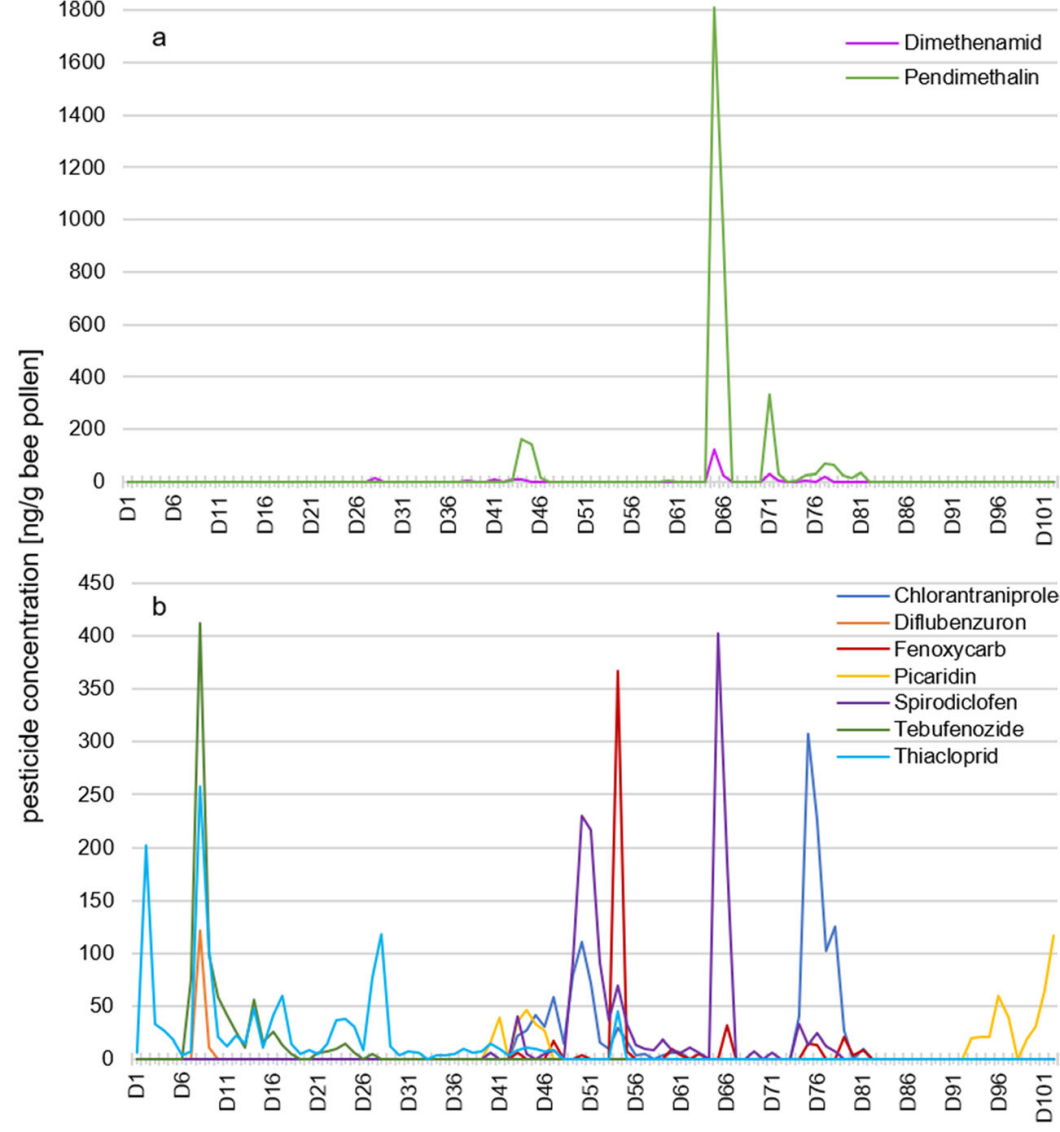
Line chart with a day-to-day progression of (**a**) herbicides with maximum concentrations and (**b**) insecticides with maximum concentrations between 100 and 400 ng/g bee pollen

*Pendimethalin (d*_*f*_ *17)* is commonly used in vegetable cultivation and crop seed conditioning. It was detected in 17 samples. The analytical recovery of pendimethalin was comparably low (55%, standard deviation 9.46%) (Table 1, Table S2). Since these results could not be reliably confirmed, the actual pendimethalin concentrations may have been underestimated by almost a factor of two. After four subsequent positive findings in early June (D43 to D46) with up to 150 ng/g pollen, pendimethalin was detected again with high abundance on D65 and D66 at 1810 and 950 ng/g pollen. After a gap of 4 days, pendimethalin was detected again between D71 and D81 (maximum concentration of 330 ng/g pollen) (Fig. 3a). *Dimethenamid (d*_*f*_ *11)* was detected in five unconnected daily pollen samples exhibiting lower concentrations in May, followed by two daily samples from June (D65 and D66) showing a maximum concentration of 120 ng/g pollen.

### Insecticides

In total, 12 insecticides were detected in all analyzed samples. Seven insecticides were observed with maximum concentrations between 100 and 415 ng/g pollen.

*Tebufenozide (d*_*f*_ *18)* was detected in 18 daily pollen samples. The first period lasted from D7 to D18 (maximum concentration of 410 ng/g pollen on D8). Two days later, tebufenozide was detected again but at much lower concentrations between D21 and D27, except one sample (Fig. 3b). Also, in this case, the period of persistence lasted for about 10 days.

The inhibitor of lipid biosynthesis s*pirodiclofen (d*_*f*_*29)* is mainly used as an acaricide and insecticide in fruit cultivation along with vegetable cultivation and crop seed treatment and is classified as hazardous to bees (Table 1). In contrast to a reported recovery rate of 70% in potatoes (Attallah et al. 2012), this study showed a calculated recovery rate of only 4% in pollen samples (61% standard deviation). These measured spirodiclofen concentrations were not considered reliable for a detailed evaluation, and only those periods in which spirodiclofen was detected were reported. Spirodiclofen was detected between D40 and D47 and then again between D49 and D63 and after one negative sample, with highest concentration measured between D65 and D66. Between D74 and D78, spirodiclofen was detected at lower level on 5 days (Fig. 3b).

*Fenoxycarb (d*_*f*_ *14)* was detected in 14 inconsecutive day samples between D43 until D81; its concentrations were typically low, except for a maximum concentration of 370 ng/g pollen at D53. Fenoxycarb is an insect growth regulator used to control scale insects on fruits or other plants and is not approved for application to blooming crops in Germany (Pesticide Properties DataBase - PPDB 2020), because it is classified hazardous to bees and can have negative effects on bee brood (Czoppelt 1991; Aupinel et al. 2007). It could contaminate pollen by drifting onto flowers and herbs in the proximity of application on non-flowering crops.

*Chlorantraniliprole (d*_*f*_ *25)* was detected between D43 and D61 at moderate to low concentrations (e.g., 110 ng/g pollen at D50) except for 1 day. Similarly, the second period lasted from D74 to D81 and featured one daily pollen sample with a maximum value of 310 ng/g pollen on D75 (Fig. 3b, Table 1). The neonicotinoid *thiacloprid (d*_*f*_ *47)* was one of the most frequently detected pesticides. Apart from one sample, it was continuously present in daily pollen samples from D1 to D49. Only for 2 days in April (D2 and D8), *thiacloprid* concentrations exceeded 200 ng/g pollen. The chitin synthesis inhibitor *diflubenzuron (d*_*f*_ *2)* is not approved for application to blooming crops in Germany and was detected in two daily samples from D7 to D8, displaying widely varying concentrations of 120 and 10 ng/g pollen, respectively. Finally, *picaridin (d*_*f*_ *15)* which is generally used as an insect repellent by humans and not as an agricultural pesticide was detected at low levels between D40 and D46. The maximum concentration of 120 ng/g pollen picaridin was determined during the last days of the study, referring to the period from D93 to D102 (Table S3). The beekeeper who collected the daily pollen samples could have been the unintended source of the picaridin contamination in the pollen samples, because he confirmed utilization of a potentially relevant insect repellent during summer.

Further five insecticides were detected with comparable low concentrations of < 100 ng/g pollen. *Fenpyroximate (d*_*f*_ *15)*, *flonicamid (d*_*f*_ *19)*, *acetamiprid (d*_*f*_ *11)*, and *methiocarb (d*_*f*_ *16)* showed their highest concentrations between D2 and D28, while *pirimicarb (d*_*f*_ *3)* was detected between D58 and D62 and again between D75 and D78 but only at lower concentrations (Figure S4b, Table S3).

It remained unclear whether the individual components were interrelated. An exemplary hierarchical cluster analysis (JMP® pro 15.0) was performed in order to show a possible connection between the presented insecticides (and further fungicides with concentrations < 100 ng/g pollen). Only one single day-to-day progression of the insecticides fenpyroximate and flonicamid is shown between D1–17, D53–58, and D101. There is no formulation used in Germany in which these two pesticides are applied together. For all other substances, no similar course between the day-to-day progressions could be established (Figure S5).

### Pollen hazard quotients at maximum concentrations

This study clearly demonstrates that pesticide levels in daily pollen samples varied strongly. Within a typical period of 10 days, concentrations peaked on 1 or 2 days and then leveled out. It was aimed to estimate the measured highest threat presented by the pesticide at maximum concentration (PHQmax). PHQmax values of the 29 detected pesticides ranged from 0.09 to 170 (Table 1). The peak value of 170 could be allocated to the insecticide methiocarb, although its corresponding maximum concentration was only 14 ng/g pollen. However, LD_50_ of methiocarb was determined corresponding to a very low level of 0.08 μg/bee (Table 1). Tebuconazole also showed a high PHQmax of 55, linked to the highest individual pesticide concentration determined in any of the samples. The tPHQday (calculated as the sum of all PHQs per sample and day) represents the total pesticide load per day. Altogether four samples revealed a tPHQday value between 100 and 180 (D6 to D8; D41), and 15 daily samples exceeded a value over 50 (Table 2). Thiacloprid was the most frequently trafficked pesticide in these samples but had comparably low PHQ values below 10 in all reported samples. In addition, methiocarb, myclobutanil, trifloxystrobin, boscalid, flonicamid, and fluopyram showed resulting PHQ values in more than 10 pollen samples within these days (Table S5). Methiocarb often had the highest PHQ values despite lower concentrations which thus contributed mainly to the high tPHQday values.

**Table 2:** Daily pollen samples with tPHQday values > 50, with total pesticide concentrations and their calculation

Sampling day	Total pesticide detects	tPHQ	Total pesticide conc. per sample (ng/g)	Thiacloprid (PHQ/pesticide conc. (ng/g))	Methiocarb (PHQ/pesticide conc. (ng/g))	Myclobutanil (PHQ/pesticide conc. (ng/g))	Trifloxystrobin (PHQ/pesticide conc. (ng/g))	Boscalid (PHQ/pesticide conc. (ng/g))	Flonicamid (PHQ/pesticide conc. (ng/g))	Fluopyram (PHQ/pesticide conc. (ng/g))	Tebuconazole (PHQ/pesticide conc. (ng/g))	Tebufenozide (PHQ/pesticide conc. (ng/g))	Pyrimethanil (PHQ/pesticide conc. (ng/g))	Difenoconazole (PHQ/pesticide conc. (ng/g))	Fenpyroximate (PHQ/pesticide conc. (ng/g))	Acetamiprid (PHQ/pesticide conc. (ng/g))	Cyprodinil (PHQ/pesticide conc. (ng/g))	Thiophanate-methyl (PHQ/pesticide conc. (ng/g))	Azoxystrobin (PHQ/pesticide conc. (ng/g))	Dimethenamid (PHQ/pesticide conc. (ng/g))	Dimoxystrobin (PHQ/pesticide conc. (ng/g))	Kresoxim-methyl (PHQ/pesticide conc. (ng/g))	Pyraclostrobin (PHQ/pesticide conc. (ng/g))	Diflubenzuron (PHQ/pesticide conc. (ng/g))
D2	7	90	571	11.6 201	70.1 5.61	6.77 227		0.05 7.5	0.25 25.2					0.03 4.6	0.84 99									
D5	6	51	88	1.1 18.9	49.7 3.89	0.6 20.4		0.15 25.4	0.13 13.1						0.04 4.85									
D6	5	174	53	0.2 3.99	174 13.9	0.34 11.5		0.02 3.22	0.19 19.2															
D7	10	142	387	0.4 75	138 11.0	0.35 12.0	0.06 7.0	0.08 14.0	0.35 35.0	1.23 126	1.1 92	0.75 75											0.07 7.44	
D8	13	183	1484	14.9 258	138 11.0	9.9334	0.59 65.0	0.08 13.0	0.13 13.4	0.46 47.0	0.59 49.0	4.12 412	0.52 52.0	0.23 40.0	0.57 68.0									13.3 121
D10	10	98	8837	1.2 21.0		0.68 23.0	0.44 48.0	0.04 6.0	0.12 12.0	39.6 4046	54.5 4527	0.58 58.0	0.46 46.0	0.27 48.0										
D11	10	80	6517	0.7 12.0	7.4 0.59	1.2 41.7	0.24 26.0		0.15 15.0	31 3169	38.2 3171	0.41 41.0	0.24 28.0		0.10 11.9									
D14	11	68	1851	2.3 47.1	46.1 3.69	0.37 12.5	1.5 159		0.18 17.9	9.7 994	5.9 485	0.55 55.4	0.09 8.98					0.51 58.7				0.07 7.73		
D15	8	50	827	0.7 11.4	40.9 3.27	0.15 5.0	0.35 38.4		0.09 8.55	5.2 525	2.6 217	0.16 16.4												
D16	13	67	335	2.4 41.3	61.5 4.92	0.25 8.58	0.8 88.0	0.51 84.2	0.06 5.77	0.24 24.7	0.10 8.3	0.25 25.3	0.07 6.99								0.08 6.12	0.18 10.5	0.01 19.4	
D20	7	53	77	0.5 9.13	51 4.08		0.16 17.6	0.12 19.5					0.06 5.53			0.73 10.6		0.09 9.8						
D24	11	68	330	2.2 37.5	62.6 5.0	0.26 8.79	1.2 136	0.04 6.44		0.47 48.3		0.15 14.9		0.03 6.08			0.4 47.4	0.09 10.1	0.3 7.56					
D25	9	71	249	1.8 30.7	66.6 5.33		1.1 119	0.03 5.15		0.43 44.5	0.07 5.73	0.07 6.5		0.02 4.19		0.32 4.66	0.2 22.6							
D28	11	53	787	6.8 118	40.1 3.2		2.2 238	0.04 6.71		1.9 194			0.08 8.36	0.02 3.06			1.7 191		0.3 6.49	0.12 14.1	0.05 3.61			
D41	7	166	268	0.6 10.3	162 13	0.43 14.5	0.12 13.5				1.9 156					0.68 9.83				0.08 10.0				

## Discussion

To our knowledge, we present for the first time a full range pesticide analysis of daily pollen samples, collected over an entire season in one of the largest contiguous fruit-growing regions in Germany. Evaluation of the collected data from 102 consecutive days has resulted in some general conclusions. Almost 90% of the analyzed daily pollen samples in our study showed detectable concentrations of pesticide residues ranging from one to thirteen pesticides per day sample. The 29 detected pesticides were dominated by 51% fungicides and then 41% insecticides and less than 10% herbicides. This trend is corroborated by results shown in studies by Drummond et al. (2018) and Böhme et al. (2018), although they observed comparatively lower insecticide levels (10–25%) in their samples. Our observation can be further supported by the results of another German study examining pesticide use over several seasons at different farms in Germany (Bürger et al. 2012). They could show diverse pesticide utilization on every individual farm, even during the same specific season. However, all farms showed a continuous usage of minimum two fungicide and one herbicide treatments per season. Some farms also use one to three insecticides, depending on the type of cultivation. Most maximum concentrations in our study were measured during April (D1 to D17) and the first half of May (D18 to D32) as well as during the second part of June (D59 to D66) (Figs. 2 a and b and 3 a and b). Fungicides and insecticides were detected throughout the whole study from D1 to D102 (April to July), while the two detected herbicides (pendimethalin and dimethenamid) were present only from D43 to D81. The repeated occurrence of active substances during the study could be due to applications to different fields, changes in pollen availability in the landscape, and bees visiting other places that have pesticide exposure such as home gardens or roadside maintenance. Due to incorrect use or drift, pesticides can also find their way into other matrices as well. Another German study detected pesticide residues in surface water in an area where 41% of the landscape is used for crop cultivation. They detected pesticide residues in water samples over an entire year, with seasonal differences in pesticide concentrations between 0.05 and 14 μg/L (Müller et al. 2002). These results confirm that pesticides cannot only be found in pollen-producing plants during a flowering season, but, moreover they become spread across the environment and thus present a permanent risk. Three pesticides (diflubenzuron, fenoxycarb, and spirodiclofen) were classified as hazardous to bees, while at the same time diflubenzuron and fenoxycarb are not approved for the usage to blooming crops in Germany (Federal Office of Consumer Protection and Food Safety 2020). Especially fenoxycarb is known to have negative effects on the bee brood; already a concentration of 50 ng/larva has been shown to induce significant damage (Czoppelt 1991; Aupinel et al. 2007). With a maximum value of 370 ng/g pollen fenoxycarb in our analyzed samples and a daily consumption of 9.5 mg bee bread per day by a nurse bee, the potential damage imposed on larvae may be low but should not be underestimated (Rortais et al. 2005). In comparison to this, Böhme et al. (2018) detected far lower concentrations of 6 ng fenoxycarb per pollen in their samples. The contamination with these pesticides could have been caused by accidental drift from other plants or another field or as a consequence of incorrect application (Pimentel 1995; de Jong et al. 2008; Lee et al. 2011). Frequently, pesticides were detected for time periods of around 10 consecutive days, exhibiting two or three maxima values, each of which several times higher compared to the previous highest concentration. This particular course was further verified by the simultaneous detection of tebuconazole and fluopyram, reaching their highest concentrations at 4500 ng/g pollen (Fig. 2). Comparatively lower pesticide concentrations were found in pollen samples from France at their maximum of 2020 ng tau-fluvalinate per g pollen (Chauzat et al. 2006). However, in our daily collected samples, maximum concentrations approached about 60% of the fenhexamid maximum level, as seen from the literature reporting a respective maximum at 7200 ng/g pollen. This was detected in a fruit-growing area in Southern Germany (Böhme et al. 2018) and was only about 5% compared to the maximum concentrations of up to 99,000 ng chlorothalonil per g pollen measured in the USA (Mullin et al. 2010; Stoner and Eitzer 2013). Typically, the maximum values in our present study were measured within the first 5 days of detection.

**Fig. 4:**
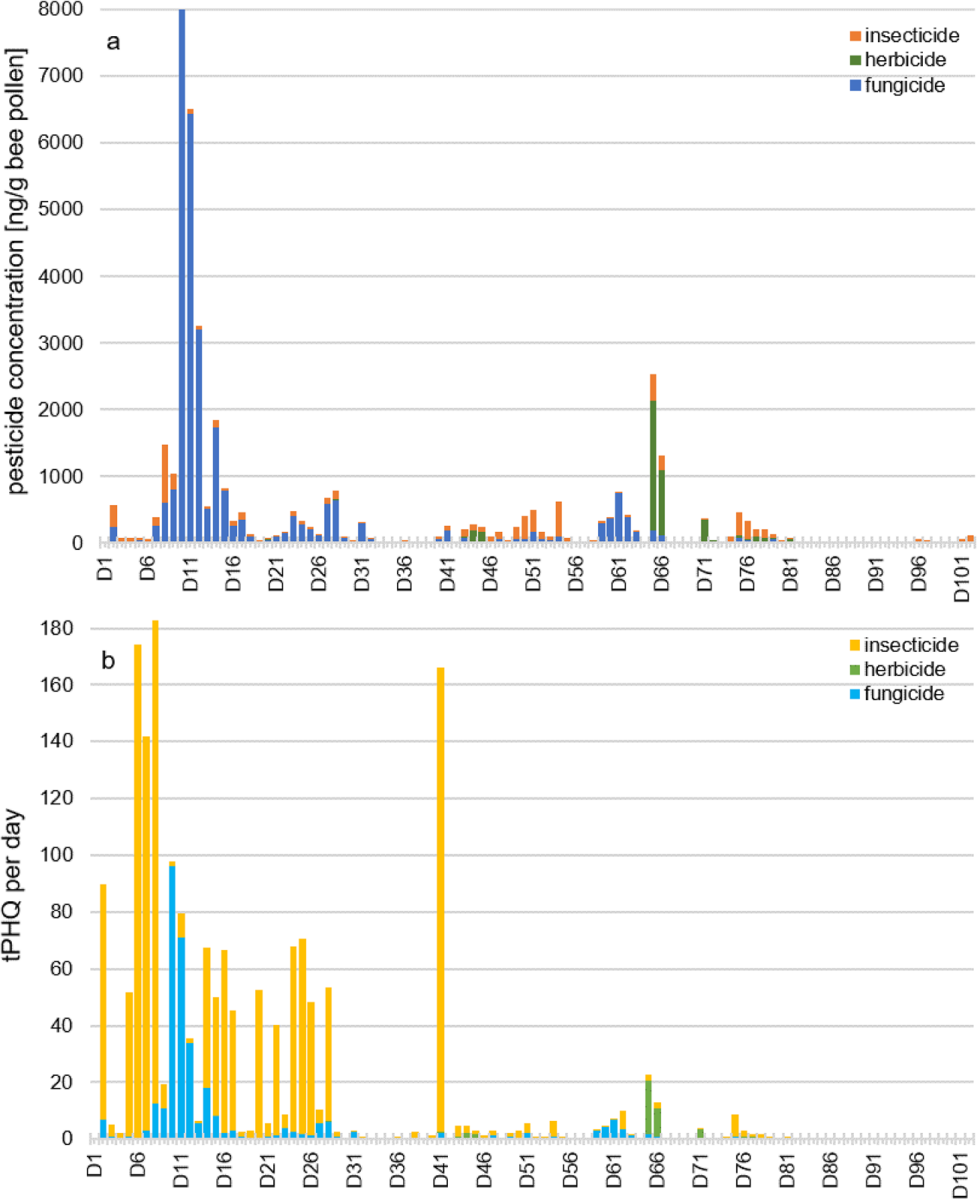
Bar chart with a day-to-day progression of (**a**) the total pesticide concentration per day and (**b**) the tPHQday values (divided into fungicide, herbicide, and insecticide class)

With respect to specific data, the data here does not indicate the occurrence acute toxic concentrations to honey bees for any of the detected pesticides. However, a general risk assessment should also include sublethal and synergistic effects of “pesticide cocktails” (Wade et al. 2019; Wernecke et al. 2019). The PHQmax values ranged between 0.09 and 170 within all observations. Fungicides and herbicides tend to show low PHQmax values between 0.08 and 55, whereas insecticides calculated higher PHQmax values to a maximum of 170 in 1-day sample. The tPHQday exceeded the relevant threshold of 50 in fifteen samples. A PHQ value of thiacloprid could be calculated in all of these samples, but with PHQ values below 10. Methiocarb showed the highest PHQ values up to 170 and, despite low pesticide concentrations, made the greatest contribution to the high PHQ values. Between D6 and D8, bees were consecutively exposed to tPHQday scores above 100 on each day, so they consumed over 500 tPHQ during this 4-day window. This is equivalent to 0.5% of the bees LD_50_ during a short 4-day window, with potentially serious implications for bee health. In comparison to the total pesticide concentrations in each daily sample, a connection between the total pesticide concentrations and the tPHQday values can only be shown in a few cases. It is notable that between D6 and D8, high tPHQday levels between 140 and 180 can be calculated, whereas the absolute pesticide concentrations only peak on days D9 to D11. However, the tPHQday values in this range are also above the relevant threshold of 50. In contrast, on D41 only a low total pesticide concentration of 270 ng/g could be measured, but the tPHQday value is over 165. With regard to which class contributes to the absolute pesticide concentration per day, it can be clearly seen that mainly fungicides followed by herbicides which are responsible for the highest pesticide concentrations. Insecticides only show a small contribution to the overall pesticide concentration (Fig. 4a). On the other hand, the class composition in the PHQ values clearly shows that insecticides in particular contribute to the high PHQ values in daily samples. In this particular case, we conclude that insecticides have a high influence on the tPHQday value, even if present at relatively low concentrations (Böhme et al. 2018; Favaro et al. 2019) (Fig. 4b, Table 2). The PHQ values presented in this study appeared lower compared to previously reported 500 to 4000 (McArt et al. 2017; Böhme et al. 2018) and even higher than 40,000, as reported mainly in other studies undertaken in the USA, where more stringent plant protection management is commonly executed (Stoner and Eitzer 2013; Favaro et al. 2019). Not included in general risk assessments of pesticide residues are other pollinators, especially wild bees. These pollinators have a small foraging range and often a short foraging period of only several days. Such insect pollinators are (i) less likely to escape from a treated field and (ii) their brood is predominantly reared directly on stored pollen contaminated by a localized pesticide mixture. This particular condition may provoke a significantly negative impact on the diversity of native pollinators (Tuell and Isaacs 2010; Mallinger et al. 2015; Park et al. 2015).

**Fig. 5:**
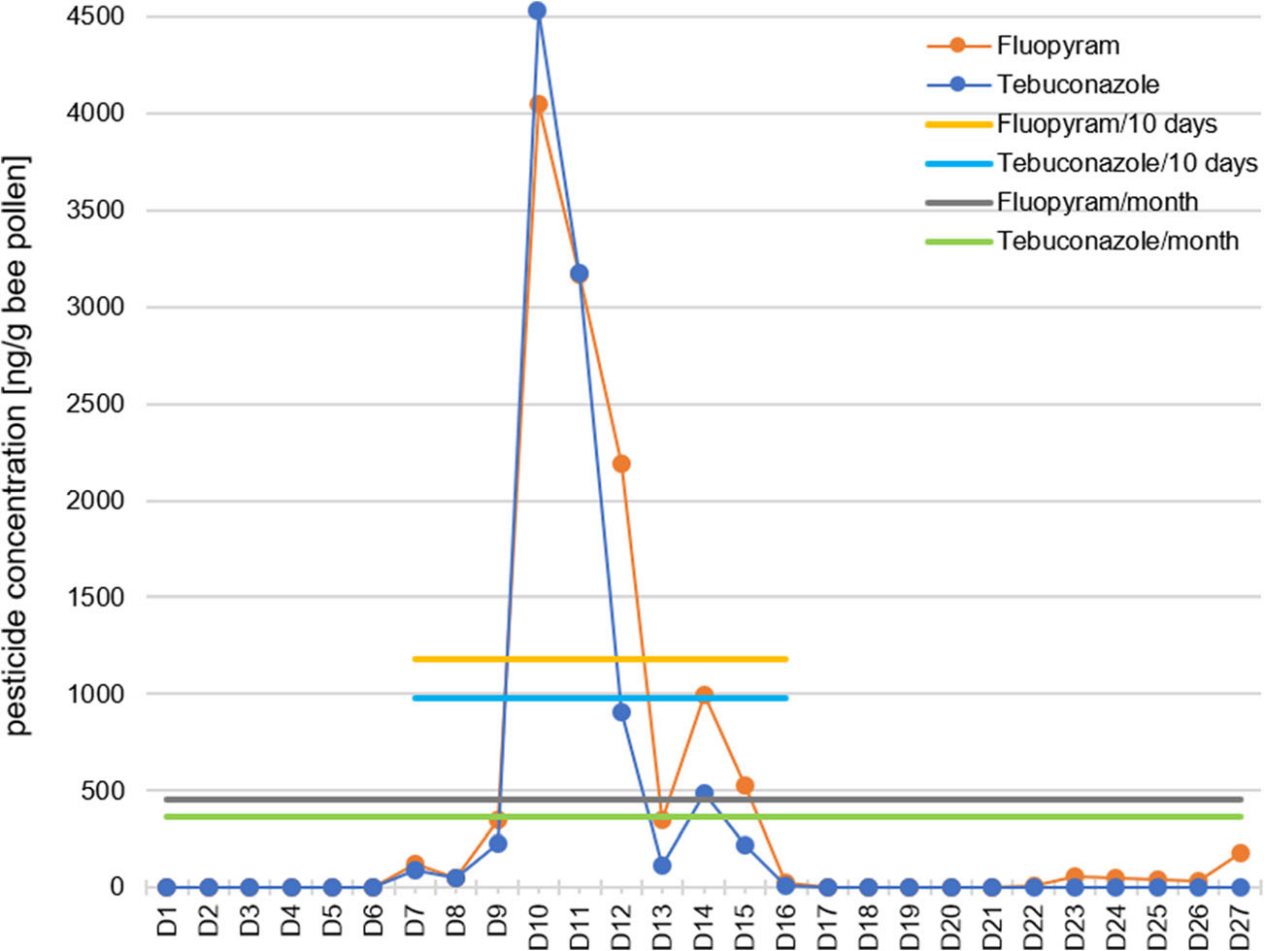
Pesticide concentrations of fluopyram and tebuconazole per day or on averaged 10 days or a full month

The occurrence of highest pesticide concentrations in only a few individual daily pollen samples may be rationalized by the assumption that in pooled pollen samples, maximum concentrations were being mitigated by dilution. For instance, fluopyram and tebuconazole which were detected together from D7 to D16 would have been expected at 1000 ng/g pollen in a respective 10-day sample pool but were only showing at 400 ng/g pollen in a pooled monthly sample (Fig. 5, Table S6). The specific dilution effect we have shown here is likely to be of general significance with respect to the evaluation of pesticide concentrations in all pooled samples. Hence, pooled pollen samples may contribute to a general underestimation of the threat to which honey bees are exposed during particular single day by a factor of between fourfold up to tenfold. This kind of potentially erroneous data evaluation turns out to be particularly important because specific maximum concentrations were found to be comparably low within the immediate surroundings of the hive. In various other situations, pesticide loads detected in daily pollen samples may also lead to the underestimation of corresponding threats to honey bees and other insects. Further extensive analytical investigations are required, across different agricultural regions, with a focus on the real daily exposure of honey bees and other pollinators to the wide range of various applied pesticide cocktails (Ostiguy et al. 2019).

## Supplementary information

ESM 1 (PDF 905 kb)

ESM 2 (PDF 467 kb)

## Data Availability

The authors confirm the data generated or analyzed during this study are included in this published article and its supplementary information files. All datasets used and analyzed during the current study is also available from the corresponding author on reasonable request.
