# Supplementary material for: Pesticide residues in daily bee pollen samples (April–July) from an intensive agricultural region in Southern Germany
Source: Environ Sci Pollut Res Int. 2021 Jan 11;28(18):22789–803. doi: 10.1007/s11356-020-12318-2 (PMC8113304; doi:10.1007/s11356-020-12318-2)
Supplement: Supplementary file 1 — (PDF 905 kb) [file 11356_2020_12318_MOESM1_ESM.pdf]

Supporting information for:

Pesticide residues in daily bee pollen samples (April-July) from an intensive agricultural region in Southern Germany

Carolin Friedle [1*] · Klaus Wallner [1] · Peter Rosenkranz[1] · Dieter Martens [2] · Walter Vetter [3]

[1] University of Hohenheim, Apicultural State Institute, Stuttgart, Germany

[2] Agricultural Research and Development Institute, Speyer, Germany

[3] University of Hohenheim, Institute of Food Chemistry (170b), Stuttgart, Germany

*carolin_friedle@uni-hohenheim.de

Table S1 Analyzed Substances with classification, LOD [ng/g] and LOQ [ng/g] as obtained by the laboratory (LUFA)

Substances	Class	LOD [ng/g]	LOQ [ng/g]
Acephate	insecticide	2	5
Acetamiprid	insecticide	1	3
Acetochlor	herbicide	1	3
Acibenzolar -s- methyl	fungicide	1	3
Alachlor	fungicide	1	3
Aldicarb	fungicide	1	3
Ametryne	herbicide	1	3
Aminocarb	insecticide	1	3
Amitraz-formamid	insecticide	1	3
Atrazin	herbicide	1	3
Azaconazole	fungicide	1	3
Azadirachtin	insecticide	2	5
Azamethiophos	insecticide	1	3
Azinphos-methyl	insecticide	1	3
Azoxystrobin	fungicide	1	3
Beflubutamid	herbicide	1	3
Bendiocarb	insecticide	2	5
Benthiavalicarb-isopropyl	fungicide	1	3
Benzovindiflupyr	fungicide	1	3
Bitertanol	fungicide	1	3
Boscalid	fungicide	1	3
Bromacil	herbicide	2	5
Butafenacil	herbicide	1	3
Butylat	herbicide	1	3
Carbaryl	insecticide	1	3
Carbendazim	fungicide	1	3
Carbetamid	herbicide	1	3
Carbofuran	insecticide	1	3
Carboxin	fungicide	1	3
Carfentrazone-ethyl	herbicide	1	3
Chlorantraniliprole	insecticide	1	3
Chlorfluazuron	insecticide	1	3
Chloridazon	herbicide	2	5
Chloroxuron	herbicide	1	3
Chlortoluron	herbicide	1	3
Cinidon-ethyl	herbicide	1	3
Clethodim	herbicide	1	3
Climbazole	fungicide	5	15
Clodinafop-propargyl	herbicide	1	3
Clofentezin	insecticide	2	5
Cloquintocet-mexyl	herbicide	2	5
Clothianidin	insecticide	1	3

Coumaphos	insecticide	1	3
Cyanazin	herbicide	1	3
Cyazofamid	fungicide	1	3
Cybutryn	fungicide	2	5
Cycloat	herbicide	1	3
Cyflufenamid	fungicide	1	3
Cymoxanil	fungicide	2	5
Cypermethrin	insecticide	1	3
Cyprodinil	fungicide	1	3
DEET	insecticide	2	5
Deltamethrin	insecticide	1	3
Demeton-S-methyl	insecticide	1	3
Demeton-S-methyl-sulfone	insecticide	1	3
Diazinon	insecticide	1	3
Diclobutrazol	fungicide	2	3
Diclofop-methyl	herbicide	2	3
Diclorvos	insecticide	2	3
Dicrotophos	herbicide	1	3
Diethofencarb	fungicide	1	3
Difenconazole	fungicide	1	3
Diflubenzuron	insecticide	1	3
Diflufenican	herbicide	1	3
Dimefox	insecticide	1	3
Dimefuron	herbicide	1	3
Dimethachlor	herbicide	1	3
Dimethenamid	herbicide	1	3
Dimethoat	insecticide	1	3
Dimethomorph	fungicide	1	3
Dimoxystrobin	fungicide	1	3
Dinotefuran	insecticide	2	5
Diuron	herbicide	1	3
EPN	insecticide	1	3
Epoxiconazole	fungicide	1	3
EPTC	herbicide	1	3
Etaconazole	fungicide	1	3
Ethidimuron	herbicide	1	3
Ethiofencarb	insecticide	2	5
Ethiofencarb-sulfone	insecticide	1	3
Ethiofencarb-sulfoxide	insecticide	1	3
Ethofumesate	herbicide	5	15
Etoxazole	insecticide	2	5
Fenamidon	fungicide	1	3
Fenchlorazol-ethyl	fungicide	2	5
Fenhexamid	fungicide	2	10
Fenothiocarb	insecticide	1	3

Fenoxycarb	insecticide	1	3
Fenpiclonil	fungicide	1	3
Fenpropimorph	fungicide	2	5
Fenpyrazamin	fungicide	1	3
Fenpyroximate	insecticide	1	3
Fenthion-oxon	insecticide	1	3
Fenthion-oxon-sulfone	insecticide	1	3
Fenthion-oxon-sulfoxide	insecticide	1	3
Fenuron	herbicide	1	3
Flonicamid	insecticide	1	3
Florasulam	herbicide	1	3
Flufenacet	herbicide	1	3
Flufenoxuron	insecticide	1	3
Flumioxazin	herbicide	3	10
Fluopicolide	fungicide	1	3
Fluopyram	fungicide	2	5
Fluoxastrobin	fungicide	1	3
Flupyradifuron	insecticide	2	5
Fluralaxyl	fungicide	1	3
Flurprimidol	plant regulator	2	5
Flurtamon	herbicide	1	3
Flutolanil	fungicide	1	3
Flutriafol	fungicide	1	3
Fluxapyroxad	fungicide	1	3
Forchlorfenuron	plant regulator	2	5
Fostthiazate	insecticide	1	3
Furathiocarb	insecticide	1	3
Halauxifen-methyl	herbicide	1	3
Halfenprox	insecticide	3	5
Hexazinone	herbicide	1	3
Hexythiazox	insecticide	2	5
Idosulfuron	herbicide	2	5
Imazalil	fungicide	3	10
Imibenconazole	fungicide	2	5
Imidacloprid	insecticide	1	3
Indaziflam	herbicide	2	5
Indoxacarb	insecticide	1	3
Iprovalicarb	fungicide	1	3
Isoprothiolan	fungicide	2	5
Isoproturon	herbicide	3	5
Isopyrazam	fungicide	1	3
Isoxaben	herbicide	1	3
Isoxadifen-ethyl	herbicide safener	1	3
Kresoxim-methyl	fungicide	2	5
Lenacil	herbicide	2	5

Linuron	herbicide	1	3
Lufenuron	insecticide	1	3
Mandipropamid	fungicide	1	3
Mefenpyr-diethyl	herbicide safener	1	3
Mepanipyrim	fungicide	1	3
Mepronil	fungicide	1	3
Mesosulfuron-methyl	herbicide	1	3
Metaflumizone	insecticide	1	3
Metalaxyl	fungicide	1	3
Metamitron	herbicide	2	5
Metazachlor	herbicide	1	3
Metconazole	fungicide	2	5
Methabenzthiazuron	herbicide	1	3
Methamidophos	insecticide	2	5
Methiocarb	insecticide	1	3
Methiocarb-sulfone	insecticide	1	3
Methiocarb-sulfoxide	insecticide	1	3
Methomyl	insecticide	1	3
Methoxyfenozide	insecticide	1	3
Metobromuron	herbicide	1	3
Metolachlor	herbicide	2	5
Metolcarb	insecticide	3	10
Metosulam	herbicide	3	10
Metoxuron	herbicide	1	3
Metrafenone	fungicide	1	3
Metribuzin	herbicide	1	3
Metsulfuron-methyl	herbicide	1	3
Molinat	herbicide	1	3
Myclobutanil	fungicide	2	5
Neburon	herbicide	2	5
Nitenpyram	herbicide	1	3
Norflurazon	herbicide	1	3
Novaluron	insecticide	1	3
Ofurace	fungicide	1	3
Omethoat	insecticide	3	10
Oxadiargyl	herbicide	1	3
Oxadiazon	herbicide	1	3
Oxadixyl	fungicide	5	15
Oxamyl	insecticide	1	3
Oxathiapiprolin	fungicide	1	3
Oxydemeton-S-methyl	insecticide	5	15
Paclobutrazol	fungicide	10	20
Pebulat	herbicide	1	3
Penconazole	fungicide	1	3
Pencycuron	fungicide	1	3

Pendimethalin	herbicide	2	5
Penflufen	fungicide	1	3
Penoxsulam	herbicide	1	3
Penthiopyrad	fungicide	1	3
Pethoxamid	herbicide	1	3
Phenisopham	herbicide	1	3
Phenmedipham	herbicide	3	10
Phosfolan	insecticide	1	3
Phosmet	insecticide	1	3
Picaridin	insecticide	5	15
Picolinafen	herbicide	2	5
Picoxystrobin	fungicide	1	3
Pirimicarb	insecticide	1	3
Pirimicarb-desmethyl	insecticide	2	5
Pirimicarb-methyl	insecticide	1	3
Prochloraz	fungicide	2	5
Promecarb	insecticide	2	5
Prometon	herbicide	1	3
Prometryn	herbicide	2	5
Propaquizafop	herbicide	1	3
Propazin	herbicide	1	3
Propetamphos	insecticide	1	3
Propham	herbicide	1	3
Propoxur	insecticide	3	10
Propyzamide	herbicide	1	3
Proquinazid	fungicide	3	10
Prosulfocarb	herbicide	1	3
Prosulfuron	herbicide	1	3
Prothioconazole	fungicide	2	5
Pyraclostrobin	fungicide	2	5
Pyraflufen-ethyl	herbicide	1	3
Pyrethrin	insecticide	2	5
Pyridalyl	insecticide	2	5
Pyrimethanil	fungicide	2	5
Pyrimidifen	insecticide	1	3
Pyriofenon	fungicide	1	3
Pyroxasulfone	herbicide	1	3
Pyroxsulam	herbicide	1	3
Quinoclamin	herbicide	2	5
Rotenon	insecticide	2	5
Saflufenacil	herbicide	2	5
Sethoxydim	herbicide	1	3
Simazin	herbicide	1	3
Spinosyn A	insecticide	3	10
Spirodiclofen	insecticide	2	5

Spiromesifen	insecticide	1	3
Spirotetramat	insecticide	1	3
Sulfentrazone	herbicide	1	3
Sulfosulfuron	herbicide	1	3
Sulfoxaflor	insecticide	1	3
Sulprofos	insecticide	2	5
Tau-Fluvalinate	insecticide	1	3
Tebuconazole	fungicide	2	5
Tebufenozide	insecticide	1	3
Tebufenpyrad	insecticide	1	3
Tebutam	herbicide	1	3
Teflubenzuron	insecticide	1	3
Tepraloxydim	herbicide	1	3
Terbumeton	herbicide	2	5
Terbuthylazine	herbicide	2	5
Terbuthylazine-desethyl	herbicide	2	5
Terbutryn	herbicide	1	3
Tetrachlorvinphos	insecticide	2	5
Thiacloprid	insecticide	1	3
Thiamethoxam	insecticide	1	3
Thifensulfuron-methyl	herbicide	1	3
Thiobencarb	herbicide	1	3
Thiodicarb	insecticide	1	3
Thiophanate-methyl	fungicide	3	10
Tolfenpyrad	insecticide	2	5
Tralkoxydim	herbicide	2	5
Triadimenol	fungicide	2	5
Triasulfuron	herbicide	1	3
Tribenuron-methyl	herbicide	2	5
Trichlorfon	insecticide	1	3
Tricyclazole	fungicide	3	10
Trifloxystrobin	fungicide	1	3
Triflumizole	fungicide	1	3
Triflusulfuron-methyl	herbicide	2	5
Triforin	fungicide	1	3
Triticonazole	fungicide	1	3
Uniconazole	plant regulator	1	3
Valifenalat	fungicide	1	3
Vamidothion	insecticide	1	3
Zoxamide	fungicide	2	5

Table S2 Recovery rates showed as mean data calculated by four blind samples

Substances	R1 [%]	R2 [%]	R3 [%]	R4 [%]	mean recovery [%]	standard deviation [%]
Acephate	67	56	56	52	58	11
Acetamiprid	106	77	74	80	84	17
Acetochlor	143	111	135	92	120	19
Acibenzolar -s- methyl	71	75	82	72	75	6
Alachlor	74	58	84	82	75	16
Aldicarb	96	90	87	72	86	12
Ametryne	78	54	66	67	66	15
Aminocarb	59	52	45	43	50	14
Amitraz-formamid	121	74	149	124	117	27
Atrazin	96	70	85	82	83	13
Azaconazole	96	62	93	88	85	19
Azadirachtin	137	119	115	133	126	9
Azamethiophos	111	89	106	134	110	17
Azinphos-methyl	101	90	105	110	101	8
Azoxystrobin	122	108	133	128	123	9
Beflubutamid	127	130	115	126	124	5
Bendiocarb	119	53	133	117	105	34
Benthiavalicarb-isopropyl	124	97	119	119	115	10
Benzovindiflupyr	126	108	136	119	122	9
Bitertanol	101	93	106	102	101	6
Boscalid	110	90	115	103	105	10
Bromacil	91	63	82	76	78	15
Butafenacil	155	125	158	180	154	15
Butylat	56	58	49	44	52	13
Carbaryl	85	99	94	66	86	17
Carbendazim	49	44	43	39	44	9
Carbetamid	82	82	119	85	92	20
Carbofuran	105	96	108	105	103	5
Carboxin	90	85	77	70	80	11
Carfentrazone-ethyl	159	117	142	125	136	14
Chlorantraniliprole	127	113	123	117	120	5
Chlorfluazuron	79	81	78	76	78	3
Chloridazon	78	62	74	71	71	10
Chloroxuron	140	91	115	88	109	22
Chlortoluron	91	88	92	67	84	14
Cinidon-ethyl	82	81	90	79	83	6
Clethodim	52	51	46	51	50	5
Climbazole	125	73	60	61	80	39
Clodinafop-propargyl	129	110	119	110	117	8
Clofentezin	70	59	63	54	62	11
Cloquintocet-mexyl	64	55	57	55	58	7
Clothianidin	78	79	81	69	77	7

Coumaphos	113	100	107	95	104	8
Cyanazin	83	72	147	91	98	34
Cyazofamid	105	87	115	110	104	12
Cybutryn	73	51	55	53	58	18
Cycloat	53	40	52	37	45	18
Cyflufenamid	103	105	111	97	104	5
Cymoxanil	84	55	80	45	66	29
Cypermethrin	73	79	80	78	78	4
Cyprodinil	25	44	27	19	29	36
DEET	105	72	111	87	94	19
Deltamethrin	90	96	104	86	94	8
Demeton-S-methyl	53	52	37	40	45	18
Demeton-S-methyl-sulfone	91	81	84	79	84	6
Diazinon	44	19	25	27	29	36
Diclobutrazol	97	82	103	95	94	10
Diclofop-methyl	89	68	82	81	80	11
Diclorvos	121	117	134	106	120	10
Dicrotophos	76	65	73	69	70	7
Diethofencarb	91	95	95	104	96	6
Difenconazole	88	86	93	81	87	6
Diflubenzuron	84	75	82	77	79	5
Diflufenican	99	86	94	95	94	6
Dimefox	60	46	48	42	49	15
Dimefuron	130	106	112	120	117	9
Dimethachlor	106	88	130	111	109	16
Dimethenamid	106	109	94	81	98	13
Dimethoat	89	67	80	74	78	12
Dimethomorph	117	110	117	120	116	4
Dimoxystrobin	114	96	122	102	108	11
Dinotefuran	74	64	64	60	65	9
Diuron	81	67	76	66	73	10
EPN	91	80	75	84	82	8
Epoxiconazole	89	77	85	73	81	9
EPTC	91	54	63	47	64	31
Etaconazole	86	78	80	63	77	13
Ethidimuron	92	72	100	79	86	15
Ethiofencarb	73	72	77	62	71	9
Ethiofencarb-sulfone	82	66	79	72	75	10
Ethiofencarb-sulfoxide	61	52	53	51	54	9
Ethofumesate	146	108	132	110	124	15
Etoxazole	70	69	75	72	71	4
Fenamidon	104	102	116	99	105	7
Fenchlorazol-ethyl	97	82	97	90	91	8
Fenhexamid	48	48	43	36	44	13
Fenothiocarb	70	85	94	61	77	19

Fenoxycarb	104	87	97	95	96	8
Fenpiclonil	37	0	79	0	29	129
Fenpropimorph	63	46	54	54	54	13
Fenpyrazamin	108	87	113	102	102	11
Fenpyroximate	78	79	85	84	82	4
Fenthion-oxon	103	78	92	84	89	12
Fenthion-oxon-sulfone	141	103	105	99	112	17
Fenthion-oxon-sulfoxide	42	26	55	48	43	29
Fenuron	107	75	91	83	89	15
Flonicamid	97	77	83	77	83	11
Florasulam	51	50	47	43	48	8
Flufenacet	120	123	125	105	118	8
Flufenoxuron	88	83	90	81	86	5
Flumioxazin	149	74	171	117	128	33
Fluopicolide	128	126	122	116	123	4
Fluopyram	124	114	132	128	125	6
Fluoxastrobin	116	110	123	117	116	5
Flupyradifuron	99	91	89	79	90	9
Fluralaxyl	95	95	122	119	107	14
Flurprimidol	93	100	86	95	94	6
Flurtamon	109	100	128	125	115	12
Flutolanil	158	118	183	173	158	18
Flutriafol	76	74	90	62	76	15
Fluxapyroxad	131	108	157	146	135	16
Forchlorfenuron	48	44	42	31	41	18
Fostthiazate	94	84	99	96	93	7
Furathiocarb	89	98	86	82	89	8
Halauxifen-methyl	108	105	125	115	113	8
Halfenprox	33	35	39	35	35	7
Hexazinone	64	49	68	70	63	15
Hexythiazox	63	61	66	64	64	3
Idosulfuron	49	68	48	62	57	17
Imazalil	31	33	40	40	36	13
Imibenconazole	80	77	84	85	81	5
Imidacloprid	87	62	82	62	73	18
Indaziflam	65	64	67	63	65	2
Indoxacarb	144	127	143	126	135	7
Iprovalicarb	111	88	125	97	105	15
Isoprothiolan	102	81	111	92	97	14
Isoproturon	99	83	74	84	85	12
Isopyrazam	114	98	113	92	104	10
Isoxaben	131	90	125	131	119	17
Isoxadifen-ethyl	142	73	117	137	117	27
Kresoxim-methyl	118	99	122	124	116	10
Lenacil	81	55	70	79	71	16

Linuron	124	83	118	90	104	20
Lufenuron	93	82	86	86	87	5
Mandipropamid	156	122	150	132	140	11
Mefenpyr-diethyl	118	118	175	117	132	22
Mepanipyrim	67	46	56	49	55	18
Mepronil	92	82	71	65	78	16
Mesosulfuron-methyl	53	63	50	46	53	13
Metaflumizone	129	106	123	121	120	8
Metalaxyl	87	88	97	81	88	7
Metamitron	78	37	73	63	63	29
Metazachlor	78	111	92	79	90	17
Metconazole	92	66	78	71	77	15
Methabenzthiazuron	71	61	84	68	71	14
Methamidophos	57	47	49	45	49	11
Methiocarb	100	75	90	113	94	17
Methiocarb-sulfone	100	64	91	106	90	21
Methiocarb-sulfoxide	76	64	70	63	68	8
Methomyl	99	79	77	73	82	14
Methoxyfenozide	147	128	103	139	129	15
Metobromuron	90	68	100	99	89	17
Metolachlor	78	82	84	83	82	3
Metolcarb	160	115	235	118	157	36
Metosulam	61	42	66	43	53	23
Metoxuron	106	75	99	71	88	20
Metrafenone	107	104	114	125	113	8
Metribuzin	61	56	76	50	61	18
Metsulfuron-methyl	42	56	42	44	46	14
Molinat	80	81	68	56	71	16
Myclobutanil	106	114	121	86	106	14
Neburon	75	67	79	72	73	7
Nitenpyram	23	7	33	54	29	67
Norflurazon	98	98	110	109	104	6
Novaluron	94	79	111	96	95	13
Ofurace	108	77	100	100	96	14
Omethoat	74	66	65	63	67	8
Oxadiargyl	145	60	161	47	103	56
Oxadiazon	108	99	109	97	103	6
Oxadixyl	90	71	111	83	89	19
Oxamyl	73	79	108	82	86	18
Oxathiapiprolin	98	98	116	95	102	10
Oxydemeton-S-methyl	58	91	37	45	58	41
Paclobutrazol	97	85	77	68	82	15
Pebulat	67	60	47	45	55	19
Penconazole	61	53	61	44	55	14
Pencycuron	80	83	78	74	79	4

Pendimethalin	57	57	49	47	53	9
Penflufen	84	82	94	85	86	6
Penoxsulam	60	45	49	43	49	15
Penthiopyrad	93	88	130	111	106	18
Pethoxamid	135	90	124	93	111	20
Phenisopham	110	100	114	94	104	9
Phenmedipham	96	97	120	89	100	14
Phosfolan	66	64	75	48	63	18
Phosmet	114	113	107	123	114	6
Picaridin	87	68	93	78	82	13
Picolinafen	113	106	96	111	107	7
Picoxystrobin	117	118	144	104	121	14
Pirimicarb	61	49	47	54	53	12
Pirimicarb-desmethyl	64	56	48	47	54	15
Pirimicarb-methyl	113	88	101	82	96	14
Prochloraz	76	71	74	66	72	6
Promecarb	89	95	108	106	100	9
Prometon	90	62	73	64	72	17
Prometryn	67	64	69	57	64	8
Propaquizafop	102	100	118	99	105	8
Propazin	77	80	76	69	75	6
Propetamphos	130	92	87	126	109	20
Propham	94	82	70	64	78	17
Propoxur	99	92	122	80	98	18
Propyzamide	91	94	109	97	98	8
Proquinazid	40	45	42	42	42	5
Prosulfocarb	67	57	65	51	60	13
Prosulfuron	38	40	41	32	38	11
Prothioconazole	70	77	81	73	75	6
Pyraclostrobin	108	88	111	95	100	11
Pyraflufen-ethyl	166	121	145	126	139	15
Pyrethrin	72	69	76	71	72	4
Pyridalyl	37	38	41	40	39	4
Pyrimethanil	59	48	52	71	57	18
Pyrimidifen	49	37	47	43	44	12
Pyriofenon	106	91	94	96	97	7
Pyroxasulfone	91	93	100	88	93	5
Pyroxsulam	59	67	77	65	67	11
Quinoclamin	53	85	167	107	103	47
Rotenon	109	107	112	97	106	6
Saflufenacil	58	52	56	63	57	8
Sethoxydim	50	50	52	45	49	6
Simazin	73	72	77	63	71	9
Spinosyn A	30	37	32	31	32	10
Spirodiclofen	2	4	8	4	4	61

Spiromesifen	97	97	123	110	107	12
Spirotetramat	162	113	127	116	129	18
Sulfentrazone	97	96	93	103	97	4
Sulfosulfuron	55	97	93	30	69	47
Sulfoxaflor	102	99	104	92	99	5
Sulprofos	70	64	66	63	66	5
Tau-Fluvalinate	89	82	98	99	92	9
Tebuconazole	101	79	91	90	90	10
Tebufenozide	108	97	125	99	107	12
Tebufenpyrad	87	74	81	77	80	7
Tebutam	77	70	81	70	74	7
Teflubenzuron	72	78	71	75	74	5
Tepraloxydim	79	78	92	70	80	11
Terbumeton	73	51	62	56	61	16
Terbuthylazine	79	74	82	56	73	16
Terbuthylazine-desethyl	78	73	82	64	74	10
Terbutryn	74	53	66	51	61	18
Tetrachlorvinphos	103	105	95	107	102	5
Thiacloprid	91	60	81	76	77	17
Thiamethoxam	98	80	86	85	87	8
Thifensulfuron-methyl	48	51	49	35	46	16
Thiobencarb	64	47	54	49	53	14
Thiodicarb	102	102	101	93	99	4
Thiophanate-methyl	92	95	113	105	101	10
Tolfenpyrad	108	123	151	162	136	18
Tralkoxydim	47	51	53	40	48	12
Triadimenol	96	80	79	81	84	10
Triasulfuron	53	51	56	48	52	6
Tribenuron-methyl	53	76	64	75	67	16
Trichlorfon	75	58	72	51	64	18
Tricyclazole	63	48	45	43	50	18
Trifloxystrobin	123	102	118	105	112	9
Triflumizole	86	83	85	75	82	6
Triflusulfuron-methyl	58	70	48	63	60	15
Triforin	151	143	176	177	162	11
Triticonazole	69	74	72	58	68	11
Uniconazole	75	65	67	62	67	8
Valifenalat	130	139	154	154	144	8
Vamidothion	89	75	79	78	80	8
Zoxamide	106	136	113	115	117	11

Table S3 Detailed pesticide residue data as obtained by the laboratory

Samples	D1	D2	D3	D4	D5	D6	D7	D8	D9	D10
Dates	2018-04-12	2018-04-15	2018-04-16	2018-04-17	2018-04-18	2018-04-19	2018-04-20	2018-04-21	2018-04-22	2018-04-23
Acetamiprid	0.00	0.02	0.00	0.00	0.00	0.00	0.02	0.00	0.00	0.00
Azoxystrobin	0.02	0.00	0.00	0.00	0.02	0.00	0.00	0.00	0.02	0.00
Boscalid	0.00	7.51	0.00	0.21	25.38	3.22	14.00	13.00	38.00	6.00
Chlorantraniliprole	0.00	0.00	0.00	0.00	0.00	0.00	0.00	0.00	0.00	0.00
Cyprodinil	0.00	0.00	0.00	0.00	0.00	0.00	0.00	0.00	0.00	0.00
Difenoconazole	0.00	4.58	0.00	5.57	0.00	0.09	0.00	40.00	29.00	48.00
Diflubenzuron	0.00	0.00	0.00	0.00	0.00	0.00	0.00	121.25	11.49	0.00
Dimethenamid	0.00	0.00	0.02	0.00	0.05	0.34	0.32	0.00	0.00	0.00
Dimoxystrobin	0.00	0.00	0.00	0.00	0.00	0.00	0.00	0.00	0.00	0.00
Fenhexamid	0.00	0.00	0.00	0.00	0.00	0.00	0.00	0.15	0.00	0.00
Fenoxycarb	0.00	0.00	0.00	0.00	0.00	0.00	0.00	0.00	0.00	0.00
Fenpyroximate	0.00	98.97	6.13	7.41	4.85	0.00	0.00	68.00	10.77	0.03
Flonicamid	0.00	25.24	12.86	7.70	13.08	19.17	35.00	13.39	13.47	12.00
Fluopyram	0.00	0.00	0.00	0.00	0.00	0.00	126.00	47.00	353.00	4046.00
Kresoxim-methyl	0.00	0.00	0.00	0.00	0.00	0.00	0.00	0.00	0.00	0.00
Methiocarb	0.00	5.61	0.19	0.00	3.98	13.88	11.00	11.00	0.00	0.00
Myclobutanil	0.00	227.06	27.87	23.69	20.39	11.49	12.00	334.00	128.00	23.00
Penconazole	0.00	0.00	0.00	0.00	0.00	0.00	0.00	0.16	0.12	0.00
Pendimethalin	0.48	0.00	0.42	0.50	0.47	0.21	0.00	0.00	0.00	1.64
Picaridin	0.00	0.00	0.00	0.00	0.00	0.00	0.00	0.00	0.00	0.00
Pirimicarb	0.00	0.00	0.19	0.07	0.19	0.09	0.00	0.06	0.01	0.00
Pyraclostrobin	0.00	0.00	0.05	0.00	0.00	0.00	7.44	0.00	0.05	0.15
Pyrimethanil	0.00	0.00	0.00	0.43	0.24	0.00	0.19	52.00	22.00	46.00
Spirodiclofen	0.00	0.00	0.00	0.00	0.00	0.00	0.00	0.00	0.00	0.00
Tebuconazole	0.38	0.42	1.62	0.00	0.00	0.00	92.00	49.00	227.00	4527.00

Tebufenozide	0.00	0.00	0.00	0.00	0.00	0.00	75.00	412.00	99.00	58.00
Thiacloprid	5.65	201.28	33.21	26.48	18.92	3.99	7.00	258.00	103.00	21.00
Thiophanate-methyl	0.00	0.00	0.00	0.00	0.00	0.00	0.00	0.00	0.00	0.36
Trifloxystrobin	0.28	0.42	0.02	0.05	0.00	0.45	7.00	65.00	15.00	48.00
Sum pesticide concentration/sample	6.82	571.11	82.59	72.11	87.55	52.93	386.98	1484.00	1049.93	8837.18

Samples	D11	D12	D13	D14	D15	D16	D17	D18	D19	D20
Dates	2018-04-24	2018-04-25	2018-04-26	2018-04-27	2018-04-28	2018-04-29	2018-04-30	2018-05-01	2018-05-02	2018-05-03
Acetamiprid	0.00	0.00	0.00	0.00	0.00	0.00	17.38	12.41	7.44	10.64
Azoxystrobin	0.00	0.00	0.18	0.25	0.23	0.46	0.18	0.16	0.00	0.10
Boscalid	0.44	0.25	0.00	0.00	0.00	84.24	200.57	52.59	11.25	19.53
Chlorantraniliprole	0.00	0.00	0.00	0.00	0.00	0.00	0.00	0.00	0.00	0.00
Cyprodinil	0.00	0.00	0.00	0.00	0.00	0.00	0.00	0.00	0.00	0.00
Difenoconazole	0.10	0.00	0.00	0.00	0.00	0.00	0.00	0.00	0.00	0.00
Diflubenzuron	0.00	0.00	0.00	0.00	0.06	0.05	0.00	0.00	0.00	0.00
Dimethenamid	0.00	0.00	0.09	0.28	0.16	0.08	0.00	0.08	0.00	0.02
Dimoxystrobin	0.00	0.00	0.00	0.00	0.00	6.12	0.00	0.00	0.00	0.00
Fenhexamid	0.00	0.00	0.00	0.00	0.00	0.00	0.00	0.00	0.00	0.00
Fenoxycarb	0.00	0.00	0.00	0.00	0.00	0.00	0.00	0.00	0.00	0.00
Fenpyroximate	11.93	0.11	0.10	0.03	0.05	0.00	0.00	0.17	0.00	0.00
Flonicamid	15.00	13.63	11.57	17.89	8.55	5.77	3.47	4.33	0.00	0.00
Fluopyram	3169.00	2192.68	350.02	993.75	525.04	24.74	1.88	0.45	0.00	0.00
Kresoxim-methyl	0.00	0.00	0.41	7.73	1.78	10.45	6.62	0.00	0.48	0.00
Methiocarb	0.59	0.00	0.00	3.69	3.27	4.92	3.01	0.00	0.15	4.08
Myclobutanil	41.67	34.55	7.89	12.47	5.00	8.58	14.60	5.00	0.00	0.00
Penconazole	0.00	0.00	0.00	0.00	0.00	0.00	0.00	0.00	0.00	0.00
Pendimethalin	0.00	0.00	0.00	0.00	0.00	0.00	0.00	0.00	0.03	0.47
Picaridin	0.00	0.00	0.00	0.00	0.00	0.00	0.33	0.00	0.00	0.00
Pirimicarb	0.19	0.02	0.00	0.04	0.24	0.00	0.19	0.00	0.05	0.48
Pyraclostrobin	0.17	0.00	0.05	0.09	0.06	19.41	48.70	15.90	0.00	0.00
Pyrimethanil	28.00	0.00	0.00	8.98	0.00	6.99	7.09	0.00	0.00	5.53
Spirodiclofen	0.00	0.07	0.00	0.00	0.00	0.00	0.00	0.00	0.00	0.00
Tebuconazole	3171.00	902.43	111.10	485.34	216.51	8.30	0.00	0.42	0.30	0.00
Tebufenozide	41.00	25.15	11.46	55.43	16.37	25.26	12.89	4.52	0.00	0.00
Thiacloprid	12.00	22.26	15.24	47.12	11.35	41.29	59.69	14.25	5.30	9.13

Thiophanate-methyl	0.24	0.00	29.67	58.72	0.00	0.00	0.00	0.23	0.00	9.80
Trifloxystrobin	26.00	68.55	21.31	159.02	38.44	88.00	77.96	25.78	11.90	17.60
Sum pesticide concentration/sample	6517.32	3259.69	559.08	1850.83	827.10	334.69	454.56	136.27	36.90	77.38

Samples	D21	D22	D23	D24	D25	D26	D27	D28	D29	D30
Dates	2018-05-04	2018-05-05	2018-05-06	2018-05-07	2018-05-08	2018-05-09	2018-05-11	2018-05-12	2018-05-13	2018-05-18
Acetamiprid	0.00	0.00	30.75	0.00	4.66	0.00	0.00	0.00	0.09	0.00
Azoxystrobin	0.43	0.40	12.35	7.56	0.00	14.42	6.47	6.49	0.49	0.09
Boscalid	43.52	54.16	4.25	6.44	5.15	0.00	6.84	6.71	13.43	7.61
Chlorantraniliprole	0.00	0.00	0.00	0.00	0.00	0.00	0.00	0.00	0.00	0.00
Cyprodinil	0.00	0.00	79.02	47.41	22.59	6.37	34.81	190.57	0.00	0.00
Difenoconazole	0.00	0.00	3.32	6.08	4.19	0.00	12.77	3.06	0.00	0.00
Diflubenzuron	0.00	0.00	0.00	0.44	0.00	0.00	0.00	0.07	0.00	0.00
Dimethenamid	0.03	0.00	0.22	0.35	0.00	0.13	0.00	14.09	0.42	0.40
Dimoxystrobin	0.14	0.04	0.05	0.20	0.00	0.00	0.00	3.61	0.15	0.00
Fenhexamid	0.00	0.00	0.00	0.00	0.00	0.00	91.31	0.00	0.00	0.00
Fenoxycarb	0.00	0.00	0.00	0.00	0.03	0.00	0.00	0.00	0.00	0.00
Fenpyroximate	0.03	0.04	0.02	0.02	0.04	0.00	4.89	0.00	4.31	4.14
Flonicamid	0.14	0.18	0.10	0.05	0.09	0.00	0.00	0.04	0.00	0.00
Fluopyram	0.32	12.65	55.72	48.28	44.46	35.61	176.02	194.12	20.32	0.00
Kresoxim-methyl	0.00	0.00	0.00	0.00	0.00	0.00	0.00	0.00	0.00	0.00
Methiocarb	0.37	3.04	0.00	5.00	5.33	3.68	0.00	3.20	0.10	0.00
Myclobutanil	0.00	9.88	0.00	8.79	0.00	0.00	0.00	0.00	0.34	0.00
Penconazole	0.00	0.00	0.00	0.00	0.00	0.00	0.18	0.26	0.00	0.00
Pendimethalin	0.00	0.00	0.00	0.00	0.00	0.00	0.00	0.00	0.10	0.00
Picaridin	0.00	0.00	0.00	0.00	0.00	0.00	0.00	0.00	0.00	0.00
Pirimicarb	0.02	0.00	0.14	0.31	0.00	0.49	0.47	0.27	0.00	0.00
Pyraclostrobin	10.26	6.99	0.00	0.00	0.38	0.29	0.00	0.05	0.00	0.43
Pyrimethanil	0.00	0.42	0.00	0.00	0.00	0.00	0.00	8.36	0.32	0.00
Spirodiclofen	0.00	0.00	0.00	0.00	0.00	0.00	0.00	0.00	0.00	0.00
Tebuconazole	0.21	0.00	0.48	0.39	5.73	0.00	0.00	0.00	0.00	0.20
Tebufenozide	5.76	7.30	9.81	14.88	6.50	0.00	4.43	0.00	0.00	0.00
Thiacloprid	4.62	14.99	37.03	37.47	30.74	8.24	77.19	118.48	12.34	4.06

Thiophanate-methyl	14.30	19.49	69.31	10.11	0.00	0.00	0.00	0.00	0.00	0.00
Trifloxystrobin	37.98	49.56	182.17	136.32	119.03	63.45	262.45	237.75	49.09	18.37
Sum pesticide concentration/sample	118.13	179.14	484.72	330.12	248.92	132.67	677.83	787.16	101.49	35.31

Samples	D31	D32	D33	D34	D35	D36	D37	D38	D39	D40
Dates	2018-05-19	2018-05-20	2018-05-21	2018-05-22	2018-05-23	2018-05-24	2018-05-25	2018-05-26	2018-05-27	2018-05-28
Acetamiprid	0.08	0.15	0.00	0.00	0.45	0.03	0.00	0.02	0.00	0.00
Azoxystrobin	0.11	0.10	0.02	0.09	0.00	9.82	0.02	0.49	0.47	0.18
Boscalid	5.01	0.00	0.00	0.00	0.00	0.15	0.00	0.00	0.00	3.09
Chlorantraniliprole	0.00	0.00	0.00	0.00	0.00	0.00	0.00	0.00	0.00	0.43
Cyprodinil	192.90	15.34	0.00	0.00	0.00	3.79	0.00	0.00	0.00	0.00
Difenoconazole	0.00	0.00	0.00	0.00	0.00	0.00	0.00	0.00	0.00	0.00
Diflubenzuron	0.00	0.00	0.00	0.00	0.00	0.00	0.00	0.00	0.00	0.00
Dimethenamid	0.43	0.00	0.00	0.33	0.00	0.00	0.00	3.39	0.00	0.00
Dimoxystrobin	0.00	0.00	0.00	0.00	0.00	0.00	0.00	0.00	0.00	0.00
Fenhexamid	0.00	0.00	0.15	0.00	0.00	0.00	0.00	0.00	0.15	22.51
Fenoxycarb	0.00	0.00	0.00	0.00	0.00	0.00	0.00	0.00	0.00	0.03
Fenpyroximate	4.51	0.46	0.00	0.00	0.00	0.00	0.00	0.00	0.00	0.17
Flonicamid	4.81	0.00	0.16	0.06	0.05	0.00	0.00	0.00	0.00	0.00
Fluopyram	6.74	0.33	0.00	0.00	0.00	0.33	0.00	0.00	0.00	0.00
Kresoxim-methyl	0.00	0.00	0.00	0.00	0.00	0.00	0.00	0.00	0.00	0.00
Methiocarb	0.00	0.00	0.00	0.00	0.00	0.00	0.00	0.19	0.00	0.00
Myclobutanil	0.00	0.00	0.00	0.45	3.13	0.00	0.00	0.00	0.00	0.00
Penconazole	6.22	3.27	0.00	0.00	0.39	0.27	0.00	0.48	0.32	0.00
Pendimethalin	0.00	0.45	0.12	0.07	0.04	0.00	0.00	0.00	0.00	0.00
Picaridin	0.00	0.00	0.00	0.00	0.00	0.00	0.00	0.00	0.00	16.07
Pirimicarb	0.00	0.30	0.12	0.00	0.00	0.00	0.00	0.00	0.03	0.00
Pyraclostrobin	0.38	0.06	0.00	0.00	0.00	0.00	0.00	0.00	0.00	0.00
Pyrimethanil	0.00	0.00	0.00	0.00	0.00	0.00	0.24	0.00	0.00	0.00
Spirodiclofen	0.08	0.00	0.00	0.00	0.00	0.00	0.00	0.00	0.14	5.70
Tebuconazole	0.00	0.00	0.00	0.00	0.00	0.00	0.00	0.00	0.19	0.06
Tebufenozide	0.00	0.00	0.22	0.09	0.21	0.13	0.34	0.00	0.00	0.00
Thiacloprid	7.09	6.40	0.00	3.95	3.32	4.76	10.05	5.63	7.90	14.90

Thiophanate-methyl	0.00	0.00	0.00	0.50	0.04	0.06	0.00	0.00	0.00	0.05
Trifloxystrobin	81.67	49.09	4.55	8.09	9.14	15.13	12.13	10.25	9.28	35.35
Sum pesticide concentration/sample	310.03	75.95	5.34	13.64	16.77	34.48	22.79	20.45	18.47	98.54

Samples	D41	D42	D43	D44	D45	D46	D47	D48	D49	D50
Dates	2018-05-29	2018-05-30	2018-05-31	2018-06-01	2018-06-02	2018-06-03	2018-06-04	2018-06-05	2018-06-06	2018-06-07
Acetamiprid	9.83	0.00	0.00	0.00	0.00	0.00	5.06	0.00	5.60	4.60
Azoxystrobin	0.42	0.15	0.21	0.00	0.00	0.00	0.24	0.04	0.13	0.13
Boscalid	0.00	0.00	57.47	3.80	0.00	0.00	0.00	0.19	0.00	21.37
Chlorantraniliprole	0.43	0.49	21.52	26.51	41.65	30.09	58.39	14.91	79.52	110.60
Cyprodinil	0.00	0.00	0.00	0.00	0.00	0.00	0.00	0.00	0.00	0.00
Difenoconazole	0.00	0.00	0.00	0.00	0.00	0.00	0.00	0.09	0.00	0.00
Diflubenzuron	0.00	0.00	0.00	0.00	0.00	0.00	0.00	0.00	0.00	0.00
Dimethenamid	10.00	0.00	8.27	8.70	0.00	0.00	0.40	0.19	0.11	0.23
Dimoxystrobin	0.00	0.00	0.00	0.00	0.00	0.00	0.00	0.00	0.00	0.00
Fenhexamid	0.00	0.00	12.04	0.00	0.00	0.00	0.00	0.14	0.00	0.00
Fenoxycarb	0.09	0.19	5.90	0.00	0.00	0.05	16.74	0.00	0.00	3.30
Fenpyroximate	0.00	0.00	0.02	0.00	0.00	0.00	0.00	0.00	0.00	0.04
Flonicamid	0.05	0.00	0.09	0.19	0.05	0.00	0.00	0.00	0.00	0.00
Fluopyram	0.00	0.00	0.48	0.00	0.00	0.33	15.77	7.74	24.34	11.04
Kresoxim-methyl	0.00	0.00	0.00	0.00	0.00	0.00	0.00	0.00	0.00	0.00
Methiocarb	12.99	0.00	0.23	0.15	0.00	0.00	0.00	0.00	0.00	0.00
Myclobutanil	14.51	0.00	5.00	12.56	16.65	10.86	30.45	5.00	10.15	8.41
Penconazole	0.00	0.00	3.76	4.19	3.36	0.00	0.00	0.00	3.74	0.00
Pendimethalin	0.00	0.00	6.96	163.71	143.24	16.49	0.00	0.00	0.00	0.00
Picaridin	39.34	0.00	35.51	46.65	33.25	26.29	0.00	0.00	0.00	0.00
Pirimicarb	0.41	0.38	0.00	0.00	0.00	0.00	0.43	0.07	0.00	0.20
Pyraclostrobin	0.34	0.07	0.97	0.15	0.17	0.00	0.10	0.04	0.31	0.00
Pyrimethanil	0.00	0.00	0.00	0.00	0.00	0.00	0.00	0.00	0.00	0.00
Spirodiclofen	0.00	0.40	39.80	5.26	0.00	5.35	9.00	0.00	91.20	229.93
Tebuconazole	155.84	0.13	0.00	0.00	0.00	0.00	9.78	0.00	15.77	8.61
Tebufenozide	0.00	0.24	0.00	0.00	0.00	0.00	0.00	0.49	0.00	0.21
Thiacloprid	10.32	3.72	8.08	11.09	10.39	7.09	8.66	0.00	0.00	0.00

Thiophanate-methyl	0.00	0.00	0.34	0.00	0.07	0.05	0.04	0.22	0.00	0.00
Trifloxystrobin	13.49	8.82	6.98	4.80	3.50	0.00	8.83	4.35	6.42	3.80
Sum pesticide concentration/sample	268.08	14.59	213.62	287.75	252.34	96.60	163.92	33.49	237.29	402.48

Samples	D51	D52	D53	D54	D55	D56	D57	D58	D59	D60
Dates	2018-06-08	2018-06-09	2018-06-10	2018-06-11	2018-06-12	2018-06-14	2018-06-15	2018-06-16	2018-06-17	2018-06-18
Acetamiprid	21.92	0.00	0.00	0.00	0.00	0.43	0.00	0.16	0.20	0.21
Azoxystrobin	0.16	0.00	0.03	0.02	0.04	0.00	0.00	0.07	0.00	0.02
Boscalid	3.90	0.00	0.00	0.00	0.25	0.00	0.16	0.00	0.00	0.00
Chlorantraniliprole	71.39	16.44	9.32	28.71	17.58	4.09	5.51	0.00	3.35	6.48
Cyprodinil	0.00	0.00	0.00	0.00	0.00	0.00	0.00	0.00	0.00	0.00
Difenoconazole	0.00	0.00	0.00	0.00	0.00	0.00	0.00	0.00	0.00	0.00
Diflubenzuron	0.00	0.00	0.00	0.00	0.00	0.00	0.00	0.00	0.00	0.00
Dimethenamid	0.13	0.00	0.22	0.30	0.32	0.16	0.45	0.19	0.07	0.08
Dimoxystrobin	0.00	0.00	0.00	0.00	0.00	0.00	0.00	0.00	0.00	0.00
Fenhexamid	0.00	0.00	0.21	0.00	0.00	0.24	0.00	0.00	274.10	132.53
Fenoxycarb	0.00	0.49	0.14	366.68	7.18	0.00	0.49	0.00	0.00	10.26
Fenpyroximate	0.16	0.00	0.00	15.83	11.32	6.27	5.79	0.44	0.00	0.00
Flonicamid	0.00	0.00	0.00	0.00	3.23	0.08	0.06	0.00	0.00	0.00
Fluopyram	25.89	10.52	0.00	47.98	5.20	0.00	0.00	0.00	0.00	0.00
Kresoxim-methyl	0.00	0.31	0.00	0.00	0.00	0.00	0.00	0.00	0.00	0.00
Methiocarb	0.00	0.00	0.00	0.00	0.00	0.00	0.00	0.00	0.00	0.00
Myclobutanil	29.86	0.00	6.51	10.63	8.68	0.00	0.00	0.00	8.32	43.69
Penconazole	0.00	0.00	0.00	0.00	0.00	0.00	0.00	24.46	22.09	9.69
Pendimethalin	0.00	0.00	0.00	0.00	0.00	0.00	0.00	0.00	0.00	5.49
Picaridin	0.00	0.00	0.00	0.00	0.00	0.00	0.00	0.00	0.00	0.00
Pirimicarb	0.26	0.12	0.02	0.16	0.40	0.00	0.00	0.34	0.00	0.00
Pyraclostrobin	0.00	0.07	0.00	0.00	0.00	0.00	0.00	0.00	0.00	0.00
Pyrimethanil	0.30	0.00	0.00	0.26	0.00	0.00	0.00	0.00	0.00	0.00
Spirodiclofen	216.50	91.86	36.33	69.71	33.08	13.50	9.33	8.92	17.81	9.04
Tebuconazole	22.97	0.00	0.00	18.47	0.00	0.34	0.00	0.09	0.35	0.00
Tebufenozide	0.26	0.15	0.09	0.00	0.00	0.11	0.21	0.00	0.00	0.00
Thiacloprid	0.00	0.00	0.00	44.51	0.00	0.00	0.00	0.00	0.00	0.00

Thiophanate-methyl	0.00	0.00	0.00	0.00	0.00	0.00	0.00	0.00	0.00	0.00
Trifloxystrobin	95.84	43.50	38.86	13.67	0.00	0.00	0.00	0.00	0.00	176.88
Sum pesticide concentration/sample	489.55	163.46	91.73	616.94	87.29	25.21	22.01	34.67	326.30	394.37

Samples	D61	D62	D63	D64	D65	D66	D67	D68	D69	D70
Dates	2018-06-19	2018-06-20	2018-06-21	2018-06-22	2018-06-23	2018-06-24	2018-06-25	2018-06-26	2018-06-27	2018-06-28
Acetamiprid	0.39	0.15	0.12	0.00	0.02	0.02	0.00	0.00	0.00	0.00
Azoxystrobin	0.00	0.05	0.16	0.12	0.02	0.12	0.04	0.00	0.00	0.00
Boscalid	0.22	0.00	0.41	0.00	0.00	24.83	0.16	0.00	0.00	0.00
Chlorantraniliprole	6.58	0.00	0.00	0.00	0.40	0.00	0.00	0.00	0.00	0.00
Cyprodinil	0.00	0.00	0.00	0.00	0.00	0.00	0.00	0.00	0.00	0.00
Difenoconazole	0.00	0.00	0.00	0.00	0.00	0.00	0.00	0.00	0.00	0.00
Diflubenzuron	0.00	0.00	0.00	0.00	0.00	0.00	0.00	0.00	0.00	0.00
Dimethenamid	0.04	0.06	0.03	0.00	120.72	25.46	0.00	0.02	0.00	0.00
Dimoxystrobin	0.00	0.00	0.00	0.00	0.00	0.00	0.00	0.00	0.00	0.00
Fenhexamid	19.98	0.00	9.68	0.00	0.00	11.65	0.00	0.00	0.00	0.16
Fenoxycarb	3.48	0.00	4.41	0.00	0.00	32.18	0.06	0.25	0.00	0.00
Fenpyroximate	0.00	0.00	0.00	0.13	0.16	0.33	0.00	0.02	0.00	0.00
Flonicamid	0.00	0.00	0.00	0.00	0.00	0.00	0.00	0.00	0.00	0.00
Fluopyram	0.00	0.00	0.00	0.00	0.00	0.00	0.00	0.00	0.00	0.00
Kresoxim-methyl	0.00	0.00	0.00	0.00	0.00	0.00	0.00	0.00	0.00	0.00
Methiocarb	0.00	0.00	0.00	0.00	0.00	0.00	0.00	0.00	0.00	0.00
Myclobutanil	14.66	6.72	0.00	0.00	0.00	0.00	0.00	0.28	0.00	0.00
Penconazole	4.66	0.00	0.00	0.00	0.49	0.00	0.00	0.00	0.00	0.00
Pendimethalin	0.00	0.00	0.00	0.41	1809.00	950.17	0.00	0.00	0.00	0.44
Picaridin	0.00	0.00	0.00	0.00	0.00	0.00	0.00	0.00	0.00	0.00
Pirimicarb	0.00	25.34	0.00	0.02	0.36	4.07	0.14	0.00	0.08	0.07
Pyraclostrobin	0.00	0.00	0.00	0.00	0.00	0.00	0.00	0.00	0.00	0.00
Pyrimethanil	0.00	0.00	0.00	0.00	0.00	0.00	0.00	0.00	0.00	0.00
Spirodiclofen	6.44	10.87	6.14	0.00	402.00	188.15	0.00	0.00	6.82	0.00
Tebuconazole	0.00	0.00	0.00	0.00	0.14	0.00	0.00	0.00	0.00	0.00
Tebufenozide	0.00	0.11	0.38	0.00	0.29	0.43	0.00	0.11	0.00	0.00
Thiacloprid	0.00	0.00	0.00	0.27	0.00	0.00	0.00	0.00	0.00	0.00

Thiophanate-methyl	0.00	0.00	0.00	0.00	0.00	0.00	0.00	0.00	0.00	0.00
Trifloxystrobin	706.62	388.64	155.46	12.00	197.00	82.37	3.16	3.84	3.33	0.00
Sum pesticide concentration/sample	736.07	431.94	176.77	12.96	2530.61	1319.78	3.56	4.53	10.24	0.67

Samples	D71	D72	D73	D74	D75	D76	D77	D78	D79	D80
Dates	2018-06-29	2018-06-30	2018-07-01	2018-07-02	2018-07-03	2018-07-04	2018-07-05	2018-07-07	2018-07-08	2018-07-09
Acetamiprid	0.02	0.00	0.00	0.00	0.44	0.00	0.00	0.03	0.00	0.00
Azoxystrobin	0.02	0.00	0.00	0.08	0.00	0.05	0.00	0.00	0.00	0.00
Boscalid	0.00	0.00	0.00	3.55	5.57	3.66	0.00	0.00	22.31	6.84
Chlorantraniliprole	0.00	0.00	0.00	39.64	307.43	225.62	101.88	125.51	26.68	0.00
Cyprodinil	0.00	0.00	0.00	0.00	0.00	0.00	0.00	0.00	0.00	0.00
Difenoconazole	0.00	0.00	0.00	0.00	0.00	0.00	0.00	0.00	0.00	0.00
Diflubenzuron	0.00	0.00	0.00	0.00	0.00	0.00	0.00	0.00	0.00	0.00
Dimethenamid	27.66	3.91	0.42	0.00	4.54	0.00	17.85	0.00	0.00	0.22
Dimoxystrobin	0.00	0.00	0.00	0.00	0.00	0.00	0.00	0.00	0.00	0.00
Fenhexamid	0.00	0.00	0.00	0.00	55.12	10.34	0.00	0.00	0.00	0.00
Fenoxycarb	0.00	0.00	0.00	0.00	14.43	13.35	0.00	0.00	20.97	3.90
Fenpyroximate	0.14	0.00	0.00	0.26	0.00	0.00	0.00	0.13	0.19	0.00
Flonicamid	0.00	0.00	0.00	0.00	0.00	0.00	0.00	0.00	0.00	0.00
Fluopyram	0.00	0.00	0.00	0.00	0.00	0.00	0.00	0.00	0.00	0.00
Kresoxim-methyl	0.00	0.00	0.00	0.00	0.00	0.00	0.00	0.00	0.00	0.00
Methiocarb	0.00	0.00	0.00	0.00	0.00	0.00	0.00	0.00	0.00	0.00
Myclobutanil	0.38	0.00	0.00	0.00	4.62	5.00	0.00	0.32	0.41	0.00
Penconazole	0.23	0.00	0.00	0.00	8.92	10.19	9.27	3.81	0.41	0.00
Pendimethalin	332.20	27.65	0.00	5.42	25.08	31.51	70.16	65.23	26.36	15.00
Picaridin	0.00	0.00	0.00	0.00	0.00	0.00	0.00	0.00	0.00	0.00
Pirimicarb	0.26	0.00	0.00	0.00	16.86	0.00	0.00	0.09	0.09	0.00
Pyraclostrobin	0.00	0.00	0.00	0.10	0.10	0.00	0.10	0.21	0.00	0.48
Pyrimethanil	0.00	0.00	0.00	0.00	0.00	0.00	0.00	0.00	0.00	0.00
Spirodiclofen	6.38	0.00	0.11	32.49	13.79	24.25	12.55	6.91	0.00	0.00
Tebuconazole	0.00	0.00	0.00	0.00	0.00	0.35	0.00	0.15	0.00	0.00
Tebufenozide	0.00	0.00	0.00	0.00	0.00	0.00	0.24	0.00	0.08	0.00
Thiacloprid	0.00	0.00	0.40	0.31	0.32	0.36	0.10	0.00	0.00	0.03

Thiophanate-methyl	0.00	0.00	0.00	0.00	0.00	0.00	0.00	0.00	0.00	0.00
Trifloxystrobin	0.00	8.77	0.00	12.59	7.40	8.28	0.00	3.67	36.44	0.00
Sum pesticide concentration/sample	367.29	40.32	0.92	94.43	464.60	332.95	212.15	206.15	133.95	26.48

Samples	D81	D82	D83	D84	D85	D86	D87	D88	D89	D90
Dates	2018-07-10	2018-07-11	2018-07-12	2018-07-13	2018-07-14	2018-07-15	2018-07-16	2018-07-17	2018-07-18	2018-07-19
Acetamiprid	0.00	0.00	0.00	0.00	0.00	0.00	0.00	0.00	0.00	0.00
Azoxystrobin	0.00	0.00	0.00	0.00	0.00	0.00	0.00	0.00	0.00	0.00
Boscalid	9.25	0.00	0.00	0.00	0.22	0.00	0.00	0.00	0.00	0.00
Chlorantraniliprole	9.63	0.00	0.00	0.00	0.00	0.00	0.00	0.00	0.00	0.00
Cyprodinil	0.00	0.00	0.00	0.00	0.00	0.00	0.00	0.00	0.00	0.00
Difenoconazole	0.00	0.00	0.00	0.00	0.00	0.00	0.00	0.00	0.00	0.00
Diflubenzuron	0.00	0.00	0.00	0.00	0.00	0.00	0.00	0.00	0.00	0.00
Dimethenamid	0.27	0.02	0.00	0.00	0.00	0.00	0.00	0.00	0.00	0.00
Dimoxystrobin	0.00	0.00	0.00	0.00	0.00	0.00	0.00	0.00	0.00	0.00
Fenhexamid	0.00	0.00	0.00	0.00	0.00	0.00	0.00	0.00	0.00	0.00
Fenoxycarb	8.55	0.00	0.00	0.00	0.00	0.00	0.00	0.00	0.00	0.00
Fenpyroximate	0.00	0.00	0.00	0.00	0.00	0.00	0.00	0.00	0.00	0.00
Flonicamid	0.00	0.08	0.00	0.00	0.00	0.00	0.00	0.00	0.00	0.00
Fluopyram	0.00	0.00	0.00	0.00	0.00	0.00	0.00	0.00	0.00	0.00
Kresoxim-methyl	0.00	0.00	0.00	0.00	0.00	0.00	0.00	0.00	0.00	0.00
Methiocarb	0.00	0.00	0.00	0.00	0.00	0.00	0.00	0.00	0.00	0.00
Myclobutanil	0.40	0.00	0.00	0.00	0.00	0.00	0.00	0.00	0.00	0.00
Penconazole	0.26	0.00	0.00	0.00	0.00	0.00	0.00	0.00	0.00	0.00
Pendimethalin	32.22	0.00	0.00	0.30	0.36	0.00	0.19	0.42	0.17	0.04
Picaridin	0.00	0.00	0.00	0.38	0.00	0.00	0.00	0.00	0.00	0.00
Pirimicarb	0.00	0.00	0.46	0.25	0.00	0.00	0.00	0.00	0.00	0.00
Pyraclostrobin	0.00	0.00	0.00	0.00	0.00	0.00	0.00	0.00	0.00	0.00
Pyrimethanil	0.00	0.00	0.00	0.00	0.00	0.00	0.00	0.00	0.00	0.00
Spirodiclofen	0.00	0.00	0.00	0.00	0.00	0.00	0.00	0.00	0.00	0.00
Tebuconazole	0.19	0.18	0.00	0.00	0.00	0.00	0.00	0.00	0.00	0.00
Tebufenozide	0.22	0.00	0.00	0.00	0.00	0.00	0.00	0.00	0.00	0.00
Thiacloprid	0.00	0.20	0.00	0.00	0.16	0.00	0.00	0.00	0.00	0.00

Thiophanate-methyl	0.00	0.00	0.00	0.00	0.00	0.00	0.00	0.00	0.00	0.00
Trifloxystrobin	19.30	0.07	0.15	0.14	0.00	0.00	0.02	0.00	0.09	0.10
Sum pesticide concentration/sample	80.29	0.55	0.62	1.08	0.74	0.00	0.21	0.42	0.26	0.14

Samples	D91	D92	D93	D94	D95	D96	D97	D98	D99	D100	D101	D102
Dates	2018-07-20	2018-07-21	2018-07-22	2018-07-23	2018-07-24	2018-07-25	2018-07-26	2018-07-27	2018-07-28	2018-07-29	2018-07-30	2018-07-31
Acetamiprid	0.00	0.00	0.00	0.00	0.00	0.00	0.00	0.00	0.00	0.00	0.00	0.00
Azoxystrobin	0.03	0.00	0.00	0.00	0.00	0.00	0.02	0.00	0.00	0.08	0.00	0.00
Boscalid	0.00	0.00	0.00	0.00	5.67	0.00	0.22	4.90	0.00	0.00	0.00	0.00
Chlorantraniliprole	0.00	0.00	0.00	0.00	0.00	0.00	0.00	0.00	0.00	0.00	0.00	0.00
Cyprodinil	0.00	0.00	0.00	0.00	0.00	0.00	0.00	0.00	0.00	0.00	0.00	0.00
Difenoconazole	0.00	0.00	0.00	0.00	0.00	0.00	0.00	0.00	0.00	0.00	0.00	0.00
Diflubenzuron	0.00	0.00	0.00	0.00	0.00	0.00	0.00	0.00	0.00	0.00	0.00	0.00
Dimethenamid	0.00	0.00	0.00	0.00	0.00	0.00	0.00	0.00	0.00	0.00	0.00	0.00
Dimoxystrobin	0.00	0.00	0.00	0.00	0.00	0.00	0.00	0.00	0.00	0.00	0.00	0.00
Fenhexamid	0.00	0.00	0.00	0.00	0.00	0.00	0.00	0.00	0.00	0.00	0.00	0.00
Fenoxycarb	0.15	0.00	0.00	0.00	0.00	0.00	0.00	0.00	0.00	0.00	0.00	0.00
Fenpyroximate	0.00	0.04	0.03	0.00	0.05	0.00	0.00	0.15	0.05	0.00	0.00	0.00
Flonicamid	0.00	0.00	0.00	0.00	0.00	0.00	0.00	0.00	0.00	0.00	0.00	0.00
Fluopyram	0.00	0.00	0.00	0.00	0.00	0.00	0.00	0.00	0.00	0.00	0.00	0.00
Kresoxim-methyl	0.00	0.00	0.00	0.00	0.00	0.00	0.00	0.00	0.00	0.00	0.00	0.00
Methiocarb	0.00	0.00	0.00	0.00	0.00	0.00	0.00	0.00	0.00	0.00	0.00	0.00
Myclobutanil	0.00	0.00	0.00	0.00	0.00	0.00	0.00	0.00	0.00	0.00	0.00	0.00
Penconazole	0.00	0.00	0.00	0.00	0.00	0.00	0.00	0.00	0.00	0.00	0.00	0.00
Pendimethalin	0.06	0.00	0.34	0.24	0.26	0.28	0.19	0.05	0.37	0.24	0.18	0.22
Picaridin	0.00	0.00	19.65	20.86	20.22	59.44	39.48	0.00	18.69	30.05	64.49	116.92
Pirimicarb	0.00	0.18	0.05	0.00	0.02	0.00	0.00	0.04	0.10	0.00	0.00	0.04
Pyraclostrobin	0.00	0.00	0.00	0.00	0.00	0.00	0.00	0.00	0.00	0.00	0.00	0.00
Pyrimethanil	0.00	0.00	0.00	0.00	0.00	0.00	0.00	0.00	0.00	0.00	0.00	0.00
Spirodiclofen	0.00	0.00	0.00	0.00	0.00	0.00	0.00	0.00	0.00	0.00	0.00	0.14
Tebuconazole	0.00	0.00	0.00	0.09	0.00	0.08	0.00	0.00	0.00	0.00	0.00	0.00
Tebufenozide	0.00	0.00	0.00	0.00	0.00	0.00	0.00	0.00	0.00	0.00	0.00	0.26
Thiacloprid	0.00	0.03	0.00	0.00	0.00	0.00	0.00	0.12	0.00	0.00	0.00	0.00

Thiophanate-methyl	0.18	0.13	0.00	0.11	0.00	0.00	0.00	0.00	0.00	0.00	0.00	0.00
Trifloxystrobin	0.04	0.47	0.07	0.00	0.00	0.00	0.00	0.00	0.00	0.00	0.00	0.00
Sum pesticide concentration/sample	0.46	0.85	20.14	21.30	26.23	59.81	39.91	5.26	19.21	30.37	64.67	117.78

Table S4 Palynological analysis exemplary counted pollen in the samples between D9 and D13 (April 22-26)

classification	counted pollen				
	D9 22.04.2018	D10 23.04.2018	D11 24.04.2018	D12 25.04.2018	D13 26.04.2018
Pinaceae_Picea sp.	0	2	2	1	5
Rosaceae_Pyrus type	17	20	15	13	10
Aceraceae_Acer sp.	1	1	2	4	20
Asteraceae_Taraxacum type	1	0	1	0	5
Rosaceae_Prunus type	72	76	78	73	55
Salicaceae_Salix sp.	9	0	0	0	0
Grossulariaceae_Ribes type	0	1	2	9	5
Summ	100	100	100	100	100

Table S5 Calculated tPHQday as the sum of all PHQs per sample and day

Samples	D1	D2	D3	D4	D5	D6	D7	D8	D9	D10
Dates	2018-04-12	2018-04-15	2018-04-16	2018-04-17	2018-04-18	2018-04-19	2018-04-20	2018-04-21	2018-04-22	2018-04-23
Acetamiprid	0.00	0.00	0.00	0.00	0.00	0.00	0.00	0.00	0.00	0.00
Azoxystrobin	0.00	0.00	0.00	0.00	0.00	0.00	0.00	0.00	0.00	0.00
Boscalid	0.00	0.05	0.00	0.00	0.15	0.02	0.08	0.08	0.23	0.04
Chlorantraniliprole	0.00	0.00	0.00	0.00	0.00	0.00	0.00	0.00	0.00	0.00
Cyprodinil	0.00	0.00	0.00	0.00	0.00	0.00	0.00	0.00	0.00	0.00
Difenoconazole	0.00	0.03	0.00	0.03	0.00	0.00	0.00	0.23	0.16	0.27
Diflubenzuron	0.00	0.00	0.00	0.00	0.00	0.00	0.00	13.32	1.26	0.00
Dimethenamid	0.00	0.00	0.00	0.00	0.00	0.00	0.00	0.00	0.00	0.00
Dimoxystrobin	0.00	0.00	0.00	0.00	0.00	0.00	0.00	0.00	0.00	0.00
Fenhexamid	0.00	0.00	0.00	0.00	0.00	0.00	0.00	0.00	0.00	0.00
Fenoxycarb	0.00	0.00	0.00	0.00	0.00	0.00	0.00	0.00	0.00	0.00
Fenpyroximate	0.00	0.84	0.05	0.06	0.04	0.00	0.00	0.57	0.09	0.00
Flonicamid	0.00	0.25	0.13	0.08	0.13	0.19	0.35	0.13	0.13	0.12
Fluopyram	0.00	0.00	0.00	0.00	0.00	0.00	1.23	0.46	3.45	39.55
Kresoxim-methyl	0.00	0.00	0.00	0.00	0.00	0.00	0.00	0.00	0.00	0.00
Methiocarb	0.00	70.12	2.40	0.00	49.69	173.53	137.50	137.50	0.00	0.00
Myclobutanil	0.00	6.70	0.82	0.70	0.60	0.34	0.35	9.85	3.78	0.68
Penconazole	0.00	0.00	0.00	0.00	0.00	0.00	0.00	0.00	0.00	0.00
Pendimethalin	0.00	0.00	0.00	0.00	0.00	0.00	0.00	0.00	0.00	0.02
Picaridin										
Pirimicarb	0.00	0.00	0.05	0.02	0.05	0.02	0.00	0.02	0.00	0.00
Pyraclostrobin	0.00	0.00	0.00	0.00	0.00	0.00	0.07	0.00	0.00	0.00
Pyrimethanil	0.00	0.00	0.00	0.00	0.00	0.00	0.00	0.52	0.22	0.46
Spirodiclofen	0.00	0.00	0.00	0.00	0.00	0.00	0.00	0.00	0.00	0.00
Tebuconazole	0.00	0.01	0.02	0.00	0.00	0.00	1.11	0.59	2.73	54.51

Tebufenozide	0.00	0.00	0.00	0.00	0.00	0.00	0.75	4.12	0.99	0.58
Thiacloprid	0.33	11.62	1.92	1.53	1.09	0.23	0.40	14.90	5.95	1.21
Thiophanate-methyl	0.00	0.00	0.00	0.00	0.00	0.00	0.00	0.00	0.00	0.00
Trifloxystrobin	0.00	0.00	0.00	0.00	0.00	0.00	0.06	0.59	0.14	0.44
Sum tPHQday	0.00	89.61	5.39	2.43	51.76	174.34	141.92	182.88	19.14	97.88

Samples	D11	D12	D13	D14	D15	D16	D17	D18	D19	D20
Dates	2018-04-24	2018-04-25	2018-04-26	2018-04-27	2018-04-28	2018-04-29	2018-04-30	2018-05-01	2018-05-02	2018-05-03
Acetamiprid	0.00	0.00	0.00	0.00	0.00	0.00	1.20	0.85	0.51	0.73
Azoxystrobin	0.00	0.00	0.01	0.01	0.01	0.02	0.01	0.01	0.00	0.00
Boscalid	0.00	0.00	0.00	0.00	0.00	0.51	1.21	0.32	0.07	0.12
Chlorantraniliprole	0.00	0.00	0.00	0.00	0.00	0.00	0.00	0.00	0.00	0.00
Cyprodinil	0.00	0.00	0.00	0.00	0.00	0.00	0.00	0.00	0.00	0.00
Difenoconazole	0.00	0.00	0.00	0.00	0.00	0.00	0.00	0.00	0.00	0.00
Diflubenzuron	0.00	0.00	0.00	0.00	0.01	0.01	0.00	0.00	0.00	0.00
Dimethenamid	0.00	0.00	0.00	0.00	0.00	0.00	0.00	0.00	0.00	0.00
Dimoxystrobin	0.00	0.00	0.00	0.00	0.00	0.08	0.00	0.00	0.00	0.00
Fenhexamid	0.00	0.00	0.00	0.00	0.00	0.00	0.00	0.00	0.00	0.00
Fenoxycarb	0.00	0.00	0.00	0.00	0.00	0.00	0.00	0.00	0.00	0.00
Fenpyroximate	0.10	0.00	0.00	0.00	0.00	0.00	0.00	0.00	0.00	0.00
Flonicamid	0.15	0.14	0.12	0.18	0.09	0.06	0.03	0.04	0.00	0.00
Fluopyram	30.98	21.43	3.42	9.71	5.13	0.24	0.02	0.00	0.00	0.00
Kresoxim-methyl	0.00	0.00	0.00	0.07	0.02	0.10	0.06	0.00	0.00	0.00
Methiocarb	7.36	0.00	0.00	46.11	40.91	61.54	37.68	0.00	1.88	50.95
Myclobutanil	1.23	1.02	0.23	0.37	0.15	0.25	0.43	0.15	0.00	0.00
Penconazole	0.00	0.00	0.00	0.00	0.00	0.00	0.00	0.00	0.00	0.00
Pendimethalin	0.00	0.00	0.00	0.00	0.00	0.00	0.00	0.00	0.00	0.00
Picaridin										
Pirimicarb	0.05	0.00	0.00	0.01	0.06	0.00	0.05	0.00	0.01	0.12
Pyraclostrobin	0.00	0.00	0.00	0.00	0.00	0.18	0.44	0.14	0.00	0.00
Pyrimethanil	0.28	0.00	0.00	0.09	0.00	0.07	0.07	0.00	0.00	0.06
Spirodiclofen	0.00	0.00	0.00	0.00	0.00	0.00	0.00	0.00	0.00	0.00
Tebuconazole	38.18	10.87	1.34	5.84	2.61	0.10	0.00	0.01	0.00	0.00
Tebufenozide	0.41	0.25	0.11	0.55	0.16	0.25	0.13	0.05	0.00	0.00
Thiacloprid	0.69	1.29	0.88	2.72	0.66	2.38	3.45	0.82	0.31	0.53

Thiophanate-methyl	0.00	0.00	0.26	0.51	0.00	0.00	0.00	0.00	0.00	0.09
Trifloxystrobin	0.24	0.62	0.19	1.45	0.35	0.80	0.71	0.23	0.11	0.16
Sum tPHQday	79.68	35.62	6.57	67.63	50.15	66.58	45.48	2.63	2.89	52.76

Samples	D21	D22	D23	D24	D25	D26	D27	D28	D29	D30
Dates	2018-05-04	2018-05-05	2018-05-06	2018-05-07	2018-05-08	2018-05-09	2018-05-11	2018-05-12	2018-05-13	2018-05-18
Acetamiprid	0.00	0.00	2.12	0.00	0.32	0.00	0.00	0.00	0.01	0.00
Azoxystrobin	0.02	0.02	0.49	0.30	0.00	0.58	0.26	0.26	0.02	0.00
Boscalid	0.26	0.33	0.03	0.04	0.03	0.00	0.04	0.04	0.08	0.05
Chlorantraniliprole	0.00	0.00	0.00	0.00	0.00	0.00	0.00	0.00	0.00	0.00
Cyprodinil	0.00	0.00	0.70	0.42	0.20	0.06	0.31	1.69	0.00	0.00
Difenoconazole	0.00	0.00	0.02	0.03	0.02	0.00	0.07	0.02	0.00	0.00
Diflubenzuron	0.00	0.00	0.00	0.05	0.00	0.00	0.00	0.01	0.00	0.00
Dimethenamid	0.00	0.00	0.00	0.00	0.00	0.00	0.00	0.12	0.00	0.00
Dimoxystrobin	0.00	0.00	0.00	0.00	0.00	0.00	0.00	0.05	0.00	0.00
Fenhexamid	0.00	0.00	0.00	0.00	0.00	0.00	0.89	0.00	0.00	0.00
Fenoxycarb	0.00	0.00	0.00	0.00	0.00	0.00	0.00	0.00	0.00	0.00
Fenpyroximate	0.00	0.00	0.00	0.00	0.00	0.00	0.04	0.00	0.04	0.03
Flonicamid	0.00	0.00	0.00	0.00	0.00	0.00	0.00	0.00	0.00	0.00
Fluopyram	0.00	0.12	0.54	0.47	0.43	0.35	1.72	1.90	0.20	0.00
Kresoxim-methyl	0.00	0.00	0.00	0.00	0.00	0.00	0.00	0.00	0.00	0.00
Methiocarb	4.60	38.04	0.00	62.55	66.59	46.01	0.00	40.05	1.21	0.00
Myclobutanil	0.00	0.29	0.00	0.26	0.00	0.00	0.00	0.00	0.01	0.00
Penconazole	0.00	0.00	0.00	0.00	0.00	0.00	0.00	0.00	0.00	0.00
Pendimethalin	0.00	0.00	0.00	0.00	0.00	0.00	0.00	0.00	0.00	0.00
Picaridin										
Pirimicarb	0.00	0.00	0.03	0.08	0.00	0.12	0.12	0.07	0.00	0.00
Pyraclostrobin	0.09	0.06	0.00	0.00	0.00	0.00	0.00	0.00	0.00	0.00
Pyrimethanil	0.00	0.00	0.00	0.00	0.00	0.00	0.00	0.08	0.00	0.00
Spirodiclofen	0.00	0.00	0.00	0.00	0.00	0.00	0.00	0.00	0.00	0.00
Tebuconazole	0.00	0.00	0.01	0.00	0.07	0.00	0.00	0.00	0.00	0.00
Tebufenozide	0.06	0.07	0.10	0.15	0.07	0.00	0.04	0.00	0.00	0.00
Thiacloprid	0.27	0.87	2.14	2.16	1.77	0.48	4.46	6.84	0.71	0.23

Thiophanate-methyl	0.12	0.17	0.60	0.09	0.00	0.00	0.00	0.00	0.00	0.00
Trifloxystrobin	0.35	0.45	1.66	1.24	1.08	0.58	2.39	2.16	0.45	0.17
Sum tPHQday	5.78	40.42	8.44	67.86	70.59	48.17	10.34	53.29	2.73	0.50

Samples	D31	D32	D33	D34	D35	D36	D37	D38	D39	D40
Dates	2018-05-19	2018-05-20	2018-05-21	2018-05-22	2018-05-23	2018-05-24	2018-05-25	2018-05-26	2018-05-27	2018-05-28
Acetamiprid	0.01	0.01	0.00	0.00	0.03	0.00	0.00	0.00	0.00	0.00
Azoxystrobin	0.00	0.00	0.00	0.00	0.00	0.39	0.00	0.02	0.02	0.01
Boscalid	0.03	0.00	0.00	0.00	0.00	0.00	0.00	0.00	0.00	0.02
Chlorantraniliprole	0.00	0.00	0.00	0.00	0.00	0.00	0.00	0.00	0.00	0.00
Cyprodinil	1.71	0.14	0.00	0.00	0.00	0.03	0.00	0.00	0.00	0.00
Difenoconazole	0.00	0.00	0.00	0.00	0.00	0.00	0.00	0.00	0.00	0.00
Diflubenzuron	0.00	0.00	0.00	0.00	0.00	0.00	0.00	0.00	0.00	0.00
Dimethenamid	0.00	0.00	0.00	0.00	0.00	0.00	0.00	0.03	0.00	0.00
Dimoxystrobin	0.00	0.00	0.00	0.00	0.00	0.00	0.00	0.00	0.00	0.00
Fenhexamid	0.00	0.00	0.00	0.00	0.00	0.00	0.00	0.00	0.00	0.22
Fenoxycarb	0.00	0.00	0.00	0.00	0.00	0.00	0.00	0.00	0.00	0.00
Fenpyroximate	0.04	0.00	0.00	0.00	0.00	0.00	0.00	0.00	0.00	0.00
Flonicamid	0.05	0.00	0.00	0.00	0.00	0.00	0.00	0.00	0.00	0.00
Fluopyram	0.07	0.00	0.00	0.00	0.00	0.00	0.00	0.00	0.00	0.00
Kresoxim-methyl	0.00	0.00	0.00	0.00	0.00	0.00	0.00	0.00	0.00	0.00
Methiocarb	0.00	0.00	0.00	0.00	0.00	0.00	0.00	2.32	0.00	0.00
Myclobutanil	0.00	0.00	0.00	0.01	0.09	0.00	0.00	0.00	0.00	0.00
Penconazole	0.06	0.03	0.00	0.00	0.00	0.00	0.00	0.00	0.00	0.00
Pendimethalin	0.00	0.00	0.00	0.00	0.00	0.00	0.00	0.00	0.00	0.00
Picaridin										
Pirimicarb	0.00	0.07	0.03	0.00	0.00	0.00	0.00	0.00	0.01	0.00
Pyraclostrobin	0.00	0.00	0.00	0.00	0.00	0.00	0.00	0.00	0.00	0.00
Pyrimethanil	0.00	0.00	0.00	0.00	0.00	0.00	0.00	0.00	0.00	0.00
Spirodiclofen	0.00	0.00	0.00	0.00	0.00	0.00	0.00	0.00	0.00	0.03
Tebuconazole	0.00	0.00	0.00	0.00	0.00	0.00	0.00	0.00	0.00	0.00
Tebufenozide	0.00	0.00	0.00	0.00	0.00	0.00	0.00	0.00	0.00	0.00
Thiacloprid	0.41	0.37	0.00	0.23	0.19	0.27	0.58	0.33	0.46	0.86

Thiophanate-methyl	0.00	0.00	0.00	0.00	0.00	0.00	0.00	0.00	0.00	0.00
Trifloxystrobin	0.74	0.45	0.04	0.07	0.08	0.14	0.11	0.09	0.08	0.32
Sum tPHQday	3.12	1.08	0.08	0.33	0.41	0.85	0.70	2.79	0.57	1.46

Samples	D41	D42	D43	D44	D45	D46	D47	D48	D49	D50
Dates	2018-05-29	2018-05-30	2018-05-31	2018-06-01	2018-06-02	2018-06-03	2018-06-04	2018-06-05	2018-06-06	2018-06-07
Acetamiprid	0.68	0.00	0.00	0.00	0.00	0.00	0.35	0.00	0.39	0.32
Azoxystrobin	0.02	0.01	0.01	0.00	0.00	0.00	0.01	0.00	0.01	0.01
Boscalid	0.00	0.00	0.35	0.02	0.00	0.00	0.00	0.00	0.00	0.13
Chlorantraniliprole	0.00	0.00	0.21	0.25	0.40	0.29	0.56	0.14	0.76	1.06
Cyprodinil	0.00	0.00	0.00	0.00	0.00	0.00	0.00	0.00	0.00	0.00
Difenoconazole	0.00	0.00	0.00	0.00	0.00	0.00	0.00	0.00	0.00	0.00
Diflubenzuron	0.00	0.00	0.00	0.00	0.00	0.00	0.00	0.00	0.00	0.00
Dimethenamid	0.08	0.00	0.07	0.07	0.00	0.00	0.00	0.00	0.00	0.00
Dimoxystrobin	0.00	0.00	0.00	0.00	0.00	0.00	0.00	0.00	0.00	0.00
Fenhexamid	0.00	0.00	0.12	0.00	0.00	0.00	0.00	0.00	0.00	0.00
Fenoxycarb	0.00	0.00	0.03	0.00	0.00	0.00	0.08	0.00	0.00	0.02
Fenpyroximate	0.00	0.00	0.00	0.00	0.00	0.00	0.00	0.00	0.00	0.00
Flonicamid	0.00	0.00	0.00	0.00	0.00	0.00	0.00	0.00	0.00	0.00
Fluopyram	0.00	0.00	0.00	0.00	0.00	0.00	0.15	0.08	0.24	0.11
Kresoxim-methyl	0.00	0.00	0.00	0.00	0.00	0.00	0.00	0.00	0.00	0.00
Methiocarb	162.43	0.00	2.86	1.82	0.00	0.00	0.00	0.00	0.00	0.00
Myclobutanil	0.43	0.00	0.15	0.37	0.49	0.32	0.90	0.15	0.30	0.25
Penconazole	0.00	0.00	0.03	0.04	0.03	0.00	0.00	0.00	0.03	0.00
Pendimethalin	0.00	0.00	0.07	1.62	1.42	0.16	0.00	0.00	0.00	0.00
Picaridin										
Pirimicarb	0.10	0.09	0.00	0.00	0.00	0.00	0.11	0.02	0.00	0.05
Pyraclostrobin	0.00	0.00	0.01	0.00	0.00	0.00	0.00	0.00	0.00	0.00
Pyrimethanil	0.00	0.00	0.00	0.00	0.00	0.00	0.00	0.00	0.00	0.00
Spirodiclofen	0.00	0.00	0.20	0.03	0.00	0.03	0.05	0.00	0.47	1.17
Tebuconazole	1.88	0.00	0.00	0.00	0.00	0.00	0.12	0.00	0.19	0.10
Tebufenozide	0.00	0.00	0.00	0.00	0.00	0.00	0.00	0.00	0.00	0.00
Thiacloprid	0.60	0.22	0.47	0.64	0.60	0.41	0.50	0.00	0.00	0.00

Thiophanate-methyl	0.00	0.00	0.00	0.00	0.00	0.00	0.00	0.00	0.00	0.00
Trifloxystrobin	0.12	0.08	0.06	0.04	0.03	0.00	0.08	0.04	0.06	0.03
Sum tPHQday	166.34	0.41	4.63	4.91	2.97	1.21	2.91	0.44	2.44	3.25

Samples	D51	D52	D53	D54	D55	D56	D57	D58	D59	D60
Dates	2018-06-08	2018-06-09	2018-06-10	2018-06-11	2018-06-12	2018-06-14	2018-06-15	2018-06-16	2018-06-17	2018-06-18
Acetamiprid	1.51	0.00	0.00	0.00	0.00	0.03	0.00	0.01	0.01	0.01
Azoxystrobin	0.01	0.00	0.00	0.00	0.00	0.00	0.00	0.00	0.00	0.00
Boscalid	0.02	0.00	0.00	0.00	0.00	0.00	0.00	0.00	0.00	0.00
Chlorantraniliprole	0.69	0.16	0.09	0.28	0.17	0.04	0.05	0.00	0.03	0.06
Cyprodinil	0.00	0.00	0.00	0.00	0.00	0.00	0.00	0.00	0.00	0.00
Difenoconazole	0.00	0.00	0.00	0.00	0.00	0.00	0.00	0.00	0.00	0.00
Diflubenzuron	0.00	0.00	0.00	0.00	0.00	0.00	0.00	0.00	0.00	0.00
Dimethenamid	0.00	0.00	0.00	0.00	0.00	0.00	0.00	0.00	0.00	0.00
Dimoxystrobin	0.00	0.00	0.00	0.00	0.00	0.00	0.00	0.00	0.00	0.00
Fenhexamid	0.00	0.00	0.00	0.00	0.00	0.00	0.00	0.00	2.69	1.30
Fenoxycarb	0.00	0.00	0.00	1.80	0.04	0.00	0.00	0.00	0.00	0.05
Fenpyroximate	0.00	0.00	0.00	0.13	0.10	0.05	0.05	0.00	0.00	0.00
Flonicamid	0.00	0.00	0.00	0.00	0.03	0.00	0.00	0.00	0.00	0.00
Fluopyram	0.25	0.10	0.00	0.47	0.05	0.00	0.00	0.00	0.00	0.00
Kresoxim-methyl	0.00	0.00	0.00	0.00	0.00	0.00	0.00	0.00	0.00	0.00
Methiocarb	0.00	0.00	0.00	0.00	0.00	0.00	0.00	0.00	0.00	0.00
Myclobutanil	0.88	0.00	0.19	0.31	0.26	0.00	0.00	0.00	0.25	1.29
Penconazole	0.00	0.00	0.00	0.00	0.00	0.00	0.00	0.22	0.20	0.09
Pendimethalin	0.00	0.00	0.00	0.00	0.00	0.00	0.00	0.00	0.00	0.05
Picaridin										
Pirimicarb	0.07	0.03	0.00	0.04	0.10	0.00	0.00	0.08	0.00	0.00
Pyraclostrobin	0.00	0.00	0.00	0.00	0.00	0.00	0.00	0.00	0.00	0.00
Pyrimethanil	0.00	0.00	0.00	0.00	0.00	0.00	0.00	0.00	0.00	0.00
Spirodiclofen	1.10	0.47	0.19	0.36	0.17	0.07	0.05	0.05	0.09	0.05
Tebuconazole	0.28	0.00	0.00	0.22	0.00	0.00	0.00	0.00	0.00	0.00
Tebufenozide	0.00	0.00	0.00	0.00	0.00	0.00	0.00	0.00	0.00	0.00
Thiacloprid	0.00	0.00	0.00	2.57	0.00	0.00	0.00	0.00	0.00	0.00

Thiophanate-methyl	0.00	0.00	0.00	0.00	0.00	0.00	0.00	0.00	0.00	0.00
Trifloxystrobin	0.87	0.40	0.35	0.12	0.00	0.00	0.00	0.00	0.00	1.61
Sum tPHQday	5.68	1.16	0.83	6.31	0.91	0.20	0.16	0.37	3.27	4.51

Samples	D61	D62	D63	D64	D65	D66	D67	D68	D69	D70
Dates	2018-06-19	2018-06-20	2018-06-21	2018-06-22	2018-06-23	2018-06-24	2018-06-25	2018-06-26	2018-06-27	2018-06-28
Acetamiprid	0.03	0.01	0.01	0.00	0.00	0.00	0.00	0.00	0.00	0.00
Azoxystrobin	0.00	0.00	0.01	0.00	0.00	0.00	0.00	0.00	0.00	0.00
Boscalid	0.00	0.00	0.00	0.00	0.00	0.15	0.00	0.00	0.00	0.00
Chlorantraniliprole	0.06	0.00	0.00	0.00	0.00	0.00	0.00	0.00	0.00	0.00
Cyprodinil	0.00	0.00	0.00	0.00	0.00	0.00	0.00	0.00	0.00	0.00
Difenoconazole	0.00	0.00	0.00	0.00	0.00	0.00	0.00	0.00	0.00	0.00
Diflubenzuron	0.00	0.00	0.00	0.00	0.00	0.00	0.00	0.00	0.00	0.00
Dimethenamid	0.00	0.00	0.00	0.00	1.02	0.22	0.00	0.00	0.00	0.00
Dimoxystrobin	0.00	0.00	0.00	0.00	0.00	0.00	0.00	0.00	0.00	0.00
Fenhexamid	0.20	0.00	0.09	0.00	0.00	0.11	0.00	0.00	0.00	0.00
Fenoxycarb	0.02	0.00	0.02	0.00	0.00	0.16	0.00	0.00	0.00	0.00
Fenpyroximate	0.00	0.00	0.00	0.00	0.00	0.00	0.00	0.00	0.00	0.00
Flonicamid	0.00	0.00	0.00	0.00	0.00	0.00	0.00	0.00	0.00	0.00
Fluopyram	0.00	0.00	0.00	0.00	0.00	0.00	0.00	0.00	0.00	0.00
Kresoxim-methyl	0.00	0.00	0.00	0.00	0.00	0.00	0.00	0.00	0.00	0.00
Methiocarb	0.00	0.00	0.00	0.00	0.00	0.00	0.00	0.00	0.00	0.00
Myclobutanil	0.43	0.20	0.00	0.00	0.00	0.00	0.00	0.01	0.00	0.00
Penconazole	0.04	0.00	0.00	0.00	0.00	0.00	0.00	0.00	0.00	0.00
Pendimethalin	0.00	0.00	0.00	0.00	17.88	9.39	0.00	0.00	0.00	0.00
Picaridin										
Pirimicarb	0.00	6.34	0.00	0.01	0.09	1.02	0.03	0.00	0.02	0.02
Pyraclostrobin	0.00	0.00	0.00	0.00	0.00	0.00	0.00	0.00	0.00	0.00
Pyrimethanil	0.00	0.00	0.00	0.00	0.00	0.00	0.00	0.00	0.00	0.00
Spirodiclofen	0.03	0.06	0.03	0.00	2.05	0.96	0.00	0.00	0.03	0.00
Tebuconazole	0.00	0.00	0.00	0.00	0.00	0.00	0.00	0.00	0.00	0.00
Tebufenozide	0.00	0.00	0.00	0.00	0.00	0.00	0.00	0.00	0.00	0.00
Thiacloprid	0.00	0.00	0.00	0.02	0.00	0.00	0.00	0.00	0.00	0.00

Thiophanate-methyl	0.00	0.00	0.00	0.00	0.00	0.00	0.00	0.00	0.00	0.00
Trifloxystrobin	6.42	3.53	1.41	0.11	1.79	0.75	0.03	0.03	0.03	0.00
Sum tPHQday	7.24	10.14	1.58	0.14	22.84	12.77	0.07	0.05	0.08	0.02

Samples	D71	D72	D73	D74	D75	D76	D77	D78	D79	D80
Dates	2018-06-29	2018-06-30	2018-07-01	2018-07-02	2018-07-03	2018-07-04	2018-07-05	2018-07-07	2018-07-08	2018-07-09
Acetamiprid	0.00	0.00	0.00	0.00	0.03	0.00	0.00	0.00	0.00	0.00
Azoxystrobin	0.00	0.00	0.00	0.00	0.00	0.00	0.00	0.00	0.00	0.00
Boscalid	0.00	0.00	0.00	0.02	0.03	0.02	0.00	0.00	0.13	0.04
Chlorantraniliprole	0.00	0.00	0.00	0.38	2.95	2.17	0.98	1.21	0.26	0.00
Cyprodinil	0.00	0.00	0.00	0.00	0.00	0.00	0.00	0.00	0.00	0.00
Difenoconazole	0.00	0.00	0.00	0.00	0.00	0.00	0.00	0.00	0.00	0.00
Diflubenzuron	0.00	0.00	0.00	0.00	0.00	0.00	0.00	0.00	0.00	0.00
Dimethenamid	0.23	0.03	0.00	0.00	0.04	0.00	0.15	0.00	0.00	0.00
Dimoxystrobin	0.00	0.00	0.00	0.00	0.00	0.00	0.00	0.00	0.00	0.00
Fenhexamid	0.00	0.00	0.00	0.00	0.54	0.10	0.00	0.00	0.00	0.00
Fenoxycarb	0.00	0.00	0.00	0.00	0.07	0.07	0.00	0.00	0.10	0.02
Fenpyroximate	0.00	0.00	0.00	0.00	0.00	0.00	0.00	0.00	0.00	0.00
Flonicamid	0.00	0.00	0.00	0.00	0.00	0.00	0.00	0.00	0.00	0.00
Fluopyram	0.00	0.00	0.00	0.00	0.00	0.00	0.00	0.00	0.00	0.00
Kresoxim-methyl	0.00	0.00	0.00	0.00	0.00	0.00	0.00	0.00	0.00	0.00
Methiocarb	0.00	0.00	0.00	0.00	0.00	0.00	0.00	0.00	0.00	0.00
Myclobutanil	0.01	0.00	0.00	0.00	0.14	0.15	0.00	0.01	0.01	0.00
Penconazole	0.00	0.00	0.00	0.00	0.08	0.09	0.08	0.03	0.00	0.00
Pendimethalin	3.28	0.27	0.00	0.05	0.25	0.31	0.69	0.64	0.26	0.15
Picaridin										
Pirimicarb	0.07	0.00	0.00	0.00	4.21	0.00	0.00	0.02	0.02	0.00
Pyraclostrobin	0.00	0.00	0.00	0.00	0.00	0.00	0.00	0.00	0.00	0.00
Pyrimethanil	0.00	0.00	0.00	0.00	0.00	0.00	0.00	0.00	0.00	0.00
Spirodiclofen	0.03	0.00	0.00	0.17	0.07	0.12	0.06	0.04	0.00	0.00
Tebuconazole	0.00	0.00	0.00	0.00	0.00	0.00	0.00	0.00	0.00	0.00
Tebufenozide	0.00	0.00	0.00	0.00	0.00	0.00	0.00	0.00	0.00	0.00
Thiacloprid	0.00	0.00	0.02	0.02	0.02	0.02	0.01	0.00	0.00	0.00

Thiophanate-methyl	0.00	0.00	0.00	0.00	0.00	0.00	0.00	0.00	0.00	0.00
Trifloxystrobin	0.00	0.08	0.00	0.11	0.07	0.08	0.00	0.03	0.33	0.00
Sum tPHQday	3.63	0.39	0.03	0.76	8.50	3.13	1.98	1.99	1.13	0.22

Samples	D81	D82	D83	D84	D85	D86	D87	D88	D89	D90
Dates	2018-07-10	2018-07-11	2018-07-12	2018-07-13	2018-07-14	2018-07-15	2018-07-16	2018-07-17	2018-07-18	2018-07-19
Acetamiprid	0.00	0.00	0.00	0.00	0.00	0.00	0.00	0.00	0.00	0.00
Azoxystrobin	0.00	0.00	0.00	0.00	0.00	0.00	0.00	0.00	0.00	0.00
Boscalid	0.06	0.00	0.00	0.00	0.00	0.00	0.00	0.00	0.00	0.00
Chlorantraniliprole	0.09	0.00	0.00	0.00	0.00	0.00	0.00	0.00	0.00	0.00
Cyprodinil	0.00	0.00	0.00	0.00	0.00	0.00	0.00	0.00	0.00	0.00
Difenoconazole	0.00	0.00	0.00	0.00	0.00	0.00	0.00	0.00	0.00	0.00
Diflubenzuron	0.00	0.00	0.00	0.00	0.00	0.00	0.00	0.00	0.00	0.00
Dimethenamid	0.00	0.00	0.00	0.00	0.00	0.00	0.00	0.00	0.00	0.00
Dimoxystrobin	0.00	0.00	0.00	0.00	0.00	0.00	0.00	0.00	0.00	0.00
Fenhexamid	0.00	0.00	0.00	0.00	0.00	0.00	0.00	0.00	0.00	0.00
Fenoxycarb	0.04	0.00	0.00	0.00	0.00	0.00	0.00	0.00	0.00	0.00
Fenpyroximate	0.00	0.00	0.00	0.00	0.00	0.00	0.00	0.00	0.00	0.00
Flonicamid	0.00	0.00	0.00	0.00	0.00	0.00	0.00	0.00	0.00	0.00
Fluopyram	0.00	0.00	0.00	0.00	0.00	0.00	0.00	0.00	0.00	0.00
Kresoxim-methyl	0.00	0.00	0.00	0.00	0.00	0.00	0.00	0.00	0.00	0.00
Methiocarb	0.00	0.00	0.00	0.00	0.00	0.00	0.00	0.00	0.00	0.00
Myclobutanil	0.01	0.00	0.00	0.00	0.00	0.00	0.00	0.00	0.00	0.00
Penconazole	0.00	0.00	0.00	0.00	0.00	0.00	0.00	0.00	0.00	0.00
Pendimethalin	0.32	0.00	0.00	0.00	0.00	0.00	0.00	0.00	0.00	0.00
Picaridin										
Pirimicarb	0.00	0.00	0.12	0.06	0.00	0.00	0.00	0.00	0.00	0.00
Pyraclostrobin	0.00	0.00	0.00	0.00	0.00	0.00	0.00	0.00	0.00	0.00
Pyrimethanil	0.00	0.00	0.00	0.00	0.00	0.00	0.00	0.00	0.00	0.00
Spirodiclofen	0.00	0.00	0.00	0.00	0.00	0.00	0.00	0.00	0.00	0.00
Tebuconazole	0.00	0.00	0.00	0.00	0.00	0.00	0.00	0.00	0.00	0.00
Tebufenozide	0.00	0.00	0.00	0.00	0.00	0.00	0.00	0.00	0.00	0.00
Thiacloprid	0.00	0.01	0.00	0.00	0.01	0.00	0.00	0.00	0.00	0.00

Thiophanate-methyl	0.00	0.00	0.00	0.00	0.00	0.00	0.00	0.00	0.00	0.00
Trifloxystrobin	0.18	0.00	0.00	0.00	0.00	0.00	0.00	0.00	0.00	0.00
Sum tPHQday	0.70	0.02	0.12	0.07	0.01	0.00	0.00	0.00	0.00	0.00

Samples	D91	D92	D93	D94	D95	D96	D97	D98	D99	D100	D101	D102
Dates	2018-07-20	2018-07-21	2018-07-22	2018-07-23	2018-07-24	2018-07-25	2018-07-26	2018-07-27	2018-07-28	2018-07-29	2018-07-30	2018-07-31
Acetamiprid	0.00	0.00	0.00	0.00	0.00	0.00	0.00	0.00	0.00	0.00	0.00	0.00
Azoxystrobin	0.00	0.00	0.00	0.00	0.00	0.00	0.00	0.00	0.00	0.00	0.00	0.00
Boscalid	0.00	0.00	0.00	0.00	0.03	0.00	0.00	0.03	0.00	0.00	0.00	0.00
Chlorantraniliprole	0.00	0.00	0.00	0.00	0.00	0.00	0.00	0.00	0.00	0.00	0.00	0.00
Cyprodinil	0.00	0.00	0.00	0.00	0.00	0.00	0.00	0.00	0.00	0.00	0.00	0.00
Difenoconazole	0.00	0.00	0.00	0.00	0.00	0.00	0.00	0.00	0.00	0.00	0.00	0.00
Diflubenzuron	0.00	0.00	0.00	0.00	0.00	0.00	0.00	0.00	0.00	0.00	0.00	0.00
Dimethenamid	0.00	0.00	0.00	0.00	0.00	0.00	0.00	0.00	0.00	0.00	0.00	0.00
Dimoxystrobin	0.00	0.00	0.00	0.00	0.00	0.00	0.00	0.00	0.00	0.00	0.00	0.00
Fenhexamid	0.00	0.00	0.00	0.00	0.00	0.00	0.00	0.00	0.00	0.00	0.00	0.00
Fenoxycarb	0.00	0.00	0.00	0.00	0.00	0.00	0.00	0.00	0.00	0.00	0.00	0.00
Fenpyroximate	0.00	0.00	0.00	0.00	0.00	0.00	0.00	0.00	0.00	0.00	0.00	0.00
Flonicamid	0.00	0.00	0.00	0.00	0.00	0.00	0.00	0.00	0.00	0.00	0.00	0.00
Fluopyram	0.00	0.00	0.00	0.00	0.00	0.00	0.00	0.00	0.00	0.00	0.00	0.00
Kresoxim-methyl	0.00	0.00	0.00	0.00	0.00	0.00	0.00	0.00	0.00	0.00	0.00	0.00
Methiocarb	0.00	0.00	0.00	0.00	0.00	0.00	0.00	0.00	0.00	0.00	0.00	0.00
Myclobutanil	0.00	0.00	0.00	0.00	0.00	0.00	0.00	0.00	0.00	0.00	0.00	0.00
Penconazole	0.00	0.00	0.00	0.00	0.00	0.00	0.00	0.00	0.00	0.00	0.00	0.00
Pendimethalin	0.00	0.00	0.00	0.00	0.00	0.00	0.00	0.00	0.00	0.00	0.00	0.00
Picaridin												
Pirimicarb	0.00	0.04	0.01	0.00	0.00	0.00	0.00	0.01	0.02	0.00	0.00	0.01
Pyraclostrobin	0.00	0.00	0.00	0.00	0.00	0.00	0.00	0.00	0.00	0.00	0.00	0.00
Pyrimethanil	0.00	0.00	0.00	0.00	0.00	0.00	0.00	0.00	0.00	0.00	0.00	0.00
Spirodiclofen	0.00	0.00	0.00	0.00	0.00	0.00	0.00	0.00	0.00	0.00	0.00	0.00
Tebuconazole	0.00	0.00	0.00	0.00	0.00	0.00	0.00	0.00	0.00	0.00	0.00	0.00
Tebufenozide	0.00	0.00	0.00	0.00	0.00	0.00	0.00	0.00	0.00	0.00	0.00	0.00
Thiacloprid	0.00	0.00	0.00	0.00	0.00	0.00	0.00	0.01	0.00	0.00	0.00	0.00

Thiophanate-methyl	0.00	0.00	0.00	0.00	0.00	0.00	0.00	0.00	0.00	0.00	0.00	0.00
Trifloxystrobin	0.00	0.00	0.00	0.00	0.00	0.00	0.00	0.00	0.00	0.00	0.00	0.00
Sum tPHQday	0.00	0.05	0.02	0.00	0.04	0.00	0.00	0.05	0.03	0.01	0.00	0.02

Table S6 Calculated pesticide concentrations in pooled samples per 10 days and month

Samples	D1	D2	D3	D4	D5	D6	D7	D8	D9	D10
Dates	2018-04-12	2018-04-15	2018-04-16	2018-04-17	2018-04-18	2018-04-19	2018-04-20	2018-04-21	2018-04-22	2018-04-23
Acetamiprid	0.00	0.02	0.00	0.00	0.00	0.00	0.02	0.00	0.00	0.00
Azoxystrobin	0.02	0.00	0.00	0.00	0.02	0.00	0.00	0.00	0.02	0.00
Boscalid	0.00	7.51	0.00	0.21	25.38	3.22	14.00	13.00	38.00	6.00
Chlorantraniliprole	0.00	0.00	0.00	0.00	0.00	0.00	0.00	0.00	0.00	0.00
Cyprodinil	0.00	0.00	0.00	0.00	0.00	0.00	0.00	0.00	0.00	0.00
Difenoconazole	0.00	4.58	0.00	5.57	0.00	0.09	0.00	40.00	29.00	48.00
Diflubenzuron	0.00	0.00	0.00	0.00	0.00	0.00	0.00	121.25	11.49	0.00
Dimethenamid	0.00	0.00	0.02	0.00	0.05	0.34	0.32	0.00	0.00	0.00
Dimoxystrobin	0.00	0.00	0.00	0.00	0.00	0.00	0.00	0.00	0.00	0.00
Fenhexamid	0.00	0.00	0.00	0.00	0.00	0.00	0.00	0.15	0.00	0.00
Fenoxycarb	0.00	0.00	0.00	0.00	0.00	0.00	0.00	0.00	0.00	0.00
Fenpyroximate	0.00	98.97	6.13	7.41	4.85	0.00	0.00	68.00	10.77	0.03
Flonicamid	0.00	25.24	12.86	7.70	13.08	19.17	35.00	13.39	13.47	12.00
Fluopyram	0.00	0.00	0.00	0.00	0.00	0.00	126.00	47.00	353.00	4046.00
Kresoxim-methyl	0.00	0.00	0.00	0.00	0.00	0.00	0.00	0.00	0.00	0.00
Methiocarb	0.00	5.61	0.19	0.00	3.98	13.88	11.00	11.00	0.00	0.00
Myclobutanil	0.00	227.06	27.87	23.69	20.39	11.49	12.00	334.00	128.00	23.00
Penconazole	0.00	0.00	0.00	0.00	0.00	0.00	0.00	0.16	0.12	0.00
Pendimethalin	0.48	0.00	0.42	0.50	0.47	0.21	0.00	0.00	0.00	1.64
Picaridin	0.00	0.00	0.00	0.00	0.00	0.00	0.00	0.00	0.00	0.00
Pirimicarb	0.00	0.00	0.19	0.07	0.19	0.09	0.00	0.06	0.01	0.00
Pyraclostrobin	0.00	0.00	0.05	0.00	0.00	0.00	7.44	0.00	0.05	0.15
Pyrimethanil	0.00	0.00	0.00	0.43	0.24	0.00	0.19	52.00	22.00	46.00
Spirodiclofen	0.00	0.00	0.00	0.00	0.00	0.00	0.00	0.00	0.00	0.00
Tebuconazole	0.38	0.42	1.62	0.00	0.00	0.00	92.00	49.00	227.00	4527.00

Tebufenozide	0.00	0.00	0.00	0.00	0.00	0.00	75.00	412.00	99.00	58.00
Thiacloprid	5.65	201.28	33.21	26.48	18.92	3.99	7.00	258.00	103.00	21.00
Thiophanate-methyl	0.00	0.00	0.00	0.00	0.00	0.00	0.00	0.00	0.00	0.36
Trifloxystrobin	0.28	0.42	0.02	0.05	0.00	0.45	7.00	65.00	15.00	48.00
Fluopyram/10 days							1183	1183	1183	1183
Tebuconazole/ 10 days							979	979	979	979
Fluopyram / month	451.95	451.95	451.95	451.95	451.95	451.95	451.95	451.95	451.95	451.95
Tebuconazole / month	362.95	362.95	362.95	362.95	362.95	362.95	362.95	362.95	362.95	362.95

Samples	D11	D12	D13	D14	D15	D16	D17	D18	D19	D20
Dates	2018-04-24	2018-04-25	2018-04-26	2018-04-27	2018-04-28	2018-04-29	2018-04-30	2018-05-01	2018-05-02	2018-05-03
Acetamiprid	0.00	0.00	0.00	0.00	0.00	0.00	17.38	12.41	7.44	10.64
Azoxystrobin	0.00	0.00	0.18	0.25	0.23	0.46	0.18	0.16	0.00	0.10
Boscalid	0.44	0.25	0.00	0.00	0.00	84.24	200.57	52.59	11.25	19.53
Chlorantraniliprole	0.00	0.00	0.00	0.00	0.00	0.00	0.00	0.00	0.00	0.00
Cyprodinil	0.00	0.00	0.00	0.00	0.00	0.00	0.00	0.00	0.00	0.00
Difenoconazole	0.10	0.00	0.00	0.00	0.00	0.00	0.00	0.00	0.00	0.00
Diflubenzuron	0.00	0.00	0.00	0.00	0.06	0.05	0.00	0.00	0.00	0.00
Dimethenamid	0.00	0.00	0.09	0.28	0.16	0.08	0.00	0.08	0.00	0.02
Dimoxystrobin	0.00	0.00	0.00	0.00	0.00	6.12	0.00	0.00	0.00	0.00
Fenhexamid	0.00	0.00	0.00	0.00	0.00	0.00	0.00	0.00	0.00	0.00
Fenoxycarb	0.00	0.00	0.00	0.00	0.00	0.00	0.00	0.00	0.00	0.00
Fenpyroximate	11.93	0.11	0.10	0.03	0.05	0.00	0.00	0.17	0.00	0.00
Flonicamid	15.00	13.63	11.57	17.89	8.55	5.77	3.47	4.33	0.00	0.00
Fluopyram	3169.00	2192.68	350.02	993.75	525.04	24.74	1.88	0.45	0.00	0.00
Kresoxim-methyl	0.00	0.00	0.41	7.73	1.78	10.45	6.62	0.00	0.48	0.00
Methiocarb	0.59	0.00	0.00	3.69	3.27	4.92	3.01	0.00	0.15	4.08
Myclobutanil	41.67	34.55	7.89	12.47	5.00	8.58	14.60	5.00	0.00	0.00
Penconazole	0.00	0.00	0.00	0.00	0.00	0.00	0.00	0.00	0.00	0.00
Pendimethalin	0.00	0.00	0.00	0.00	0.00	0.00	0.00	0.00	0.03	0.47
Picaridin	0.00	0.00	0.00	0.00	0.00	0.00	0.33	0.00	0.00	0.00
Pirimicarb	0.19	0.02	0.00	0.04	0.24	0.00	0.19	0.00	0.05	0.48
Pyraclostrobin	0.17	0.00	0.05	0.09	0.06	19.41	48.70	15.90	0.00	0.00
Pyrimethanil	28.00	0.00	0.00	8.98	0.00	6.99	7.09	0.00	0.00	5.53
Spirodiclofen	0.00	0.07	0.00	0.00	0.00	0.00	0.00	0.00	0.00	0.00
Tebuconazole	3171.00	902.43	111.10	485.34	216.51	8.30	0.00	0.42	0.30	0.00
Tebufenozide	41.00	25.15	11.46	55.43	16.37	25.26	12.89	4.52	0.00	0.00
Thiacloprid	12.00	22.26	15.24	47.12	11.35	41.29	59.69	14.25	5.30	9.13

Thiophanate-methyl	0.24	0.00	29.67	58.72	0.00	0.00	0.00	0.23	0.00	9.80
Trifloxystrobin	26.00	68.55	21.31	159.02	38.44	88.00	77.96	25.78	11.90	17.60
Fluopyram/10 days	1183	1183	1183	1183	1183	1183				
Tebuconazole/ 10 days	979	979	979	979	979	979				
Fluopyram / month	451.95	451.95	451.95	451.95	451.95	451.95	451.95	451.95	451.95	451.95
Tebuconazole / month	362.95	362.95	362.95	362.95	362.95	362.95	362.95	362.95	362.95	362.95

Samples	D21	D22	D23	D24	D25	D26	D27	Summ of 27 days	conc/month
Dates	2018-05-04	2018-05-05	2018-05-06	2018-05-07	2018-05-08	2018-05-09	2018-05-11		
Acetamiprid	0.00	0.00	30.75	0.00	4.66	0.00	0.00	83.31	3.09
Azoxystrobin	0.43	0.40	12.35	7.56	0.00	14.42	6.47	43.23	1.60
Boscalid	43.52	54.16	4.25	6.44	5.15	0.00	6.84	596.55	22.09
Chlorantraniliprole	0.00	0.00	0.00	0.00	0.00	0.00	0.00	0.00	0.00
Cyprodinil	0.00	0.00	79.02	47.41	22.59	6.37	34.81	190.20	7.04
Difenoconazole	0.00	0.00	3.32	6.08	4.19	0.00	12.77	153.70	5.69
Diflubenzuron	0.00	0.00	0.00	0.44	0.00	0.00	0.00	133.29	4.94
Dimethenamid	0.03	0.00	0.22	0.35	0.00	0.13	0.00	2.18	0.08
Dimoxystrobin	0.14	0.04	0.05	0.20	0.00	0.00	0.00	6.54	0.24
Fenhexamid	0.00	0.00	0.00	0.00	0.00	0.00	91.31	91.46	3.39
Fenoxycarb	0.00	0.00	0.00	0.00	0.03	0.00	0.00	0.03	0.00
Fenpyroximate	0.03	0.04	0.02	0.02	0.04	0.00	4.89	213.57	7.91
Flonicamid	0.14	0.18	0.10	0.05	0.09	0.00	0.00	232.67	8.62
Fluopyram	0.32	12.65	55.72	48.28	44.46	35.61	176.02	12202.62	451.95
Kresoxim-methyl	0.00	0.00	0.00	0.00	0.00	0.00	0.00	27.47	1.02
Methiocarb	0.37	3.04	0.00	5.00	5.33	3.68	0.00	82.80	3.07
Myclobutanil	0.00	9.88	0.00	8.79	0.00	0.00	0.00	955.93	35.40
Penconazole	0.00	0.00	0.00	0.00	0.00	0.00	0.18	0.46	0.02
Pendimethalin	0.00	0.00	0.00	0.00	0.00	0.00	0.00	4.22	0.16
Picaridin	0.00	0.00	0.00	0.00	0.00	0.00	0.00	0.33	0.01
Pirimicarb	0.02	0.00	0.14	0.31	0.00	0.49	0.47	3.23	0.12
Pyraclostrobin	10.26	6.99	0.00	0.00	0.38	0.29	0.00	110.01	4.07
Pyrimethanil	0.00	0.42	0.00	0.00	0.00	0.00	0.00	177.88	6.59
Spirodiclofen	0.00	0.00	0.00	0.00	0.00	0.00	0.00	0.07	0.00
Tebuconazole	0.21	0.00	0.48	0.39	5.73	0.00	0.00	9799.61	362.95
Tebufenozide	5.76	7.30	9.81	14.88	6.50	0.00	4.43	884.75	32.77
Thiacloprid	4.62	14.99	37.03	37.47	30.74	8.24	77.19	1126.45	41.72

Thiophanate-methyl	14.30	19.49	69.31	10.11	0.00	0.00	0.00	212.22	7.86
Trifloxystrobin	37.98	49.56	182.17	136.32	119.03	63.45	262.45	1521.75	56.36
Fluopyram/10 days									
Tebuconazole/ 10 days									
Fluopyram / month	451.95	451.95	451.95	451.95	451.95	451.95	451.95		
Tebuconazole / month	362.95	362.95	362.95	362.95	362.95	362.95	362.95		
