# Supplementary material for: Pesticide residues in daily bee pollen samples (April–July) from an intensive agricultural region in Southern Germany
Source: Environ Sci Pollut Res Int. 2021 Jan 11;28(18):22789–803. doi: 10.1007/s11356-020-12318-2 (PMC8113304; doi:10.1007/s11356-020-12318-2)

Supporting information for:

Pesticide residues in daily bee pollen samples (April-July) from an intensive agricultural region in Southern Germany

Carolin Friedle [1*] · Klaus Wallner [1] · Peter Rosenkranz[1] · Dieter Martens [2] · Walter Vetter [3]

[1] University of Hohenheim, Apicultural State Institute, Stuttgart, Germany

[2] Agricultural Research and Development Institute, Speyer, Germany

[3] University of Hohenheim, Institute of Food Chemistry (170b), Stuttgart, Germany

*carolin_friedle@uni-hohenheim.de

Fig. S1 Exemplary installed bee pollen trap on a bee hive

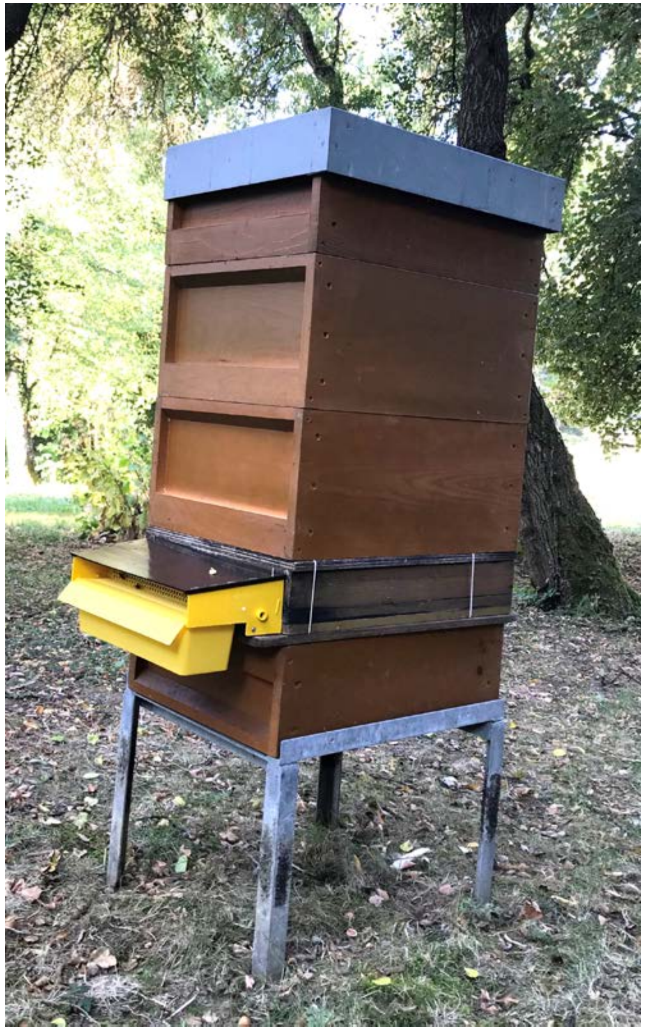

Fig. S2 Detection frequency (*d_f*) of individual pesticides in 102 daily pollen samples

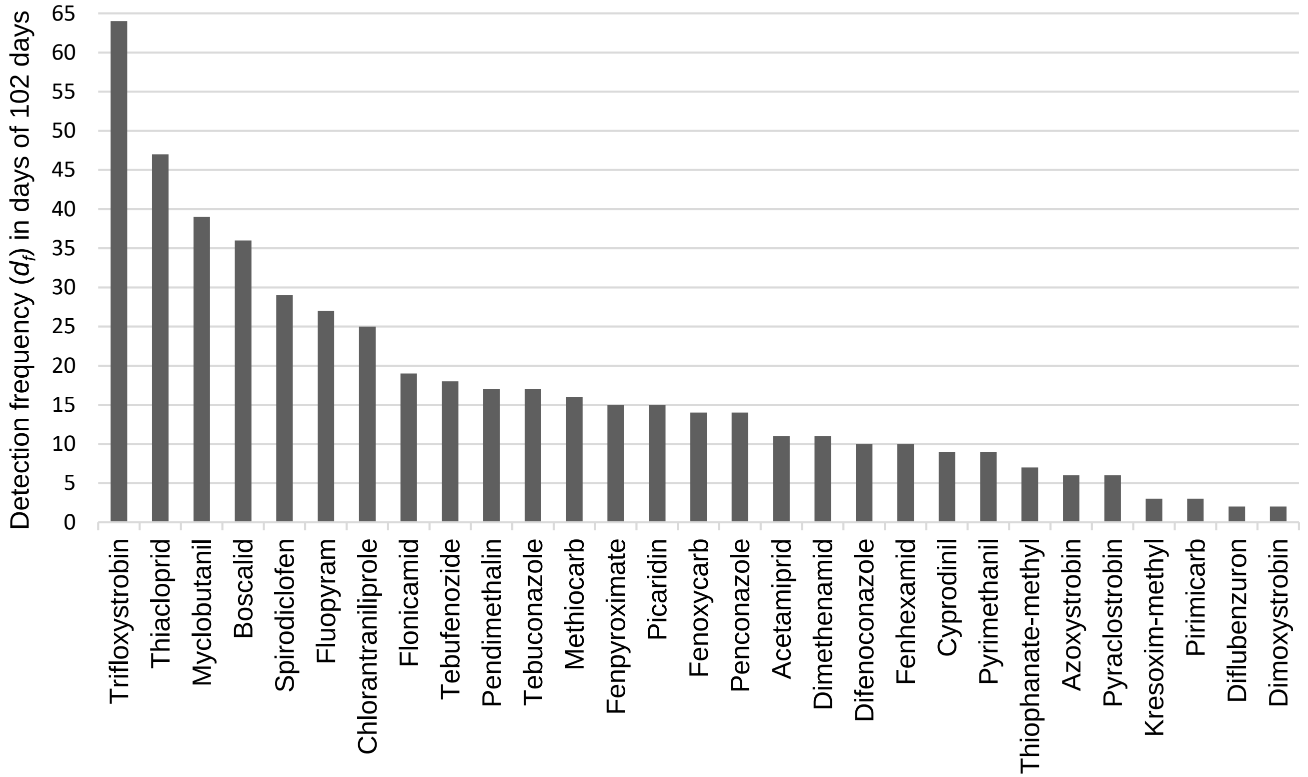

Fig. S3 Concentrations (ng/g bee pollen) of tebuconazole and fluopyram in daily pollen samples collected between D7-D16 (April 20-29)

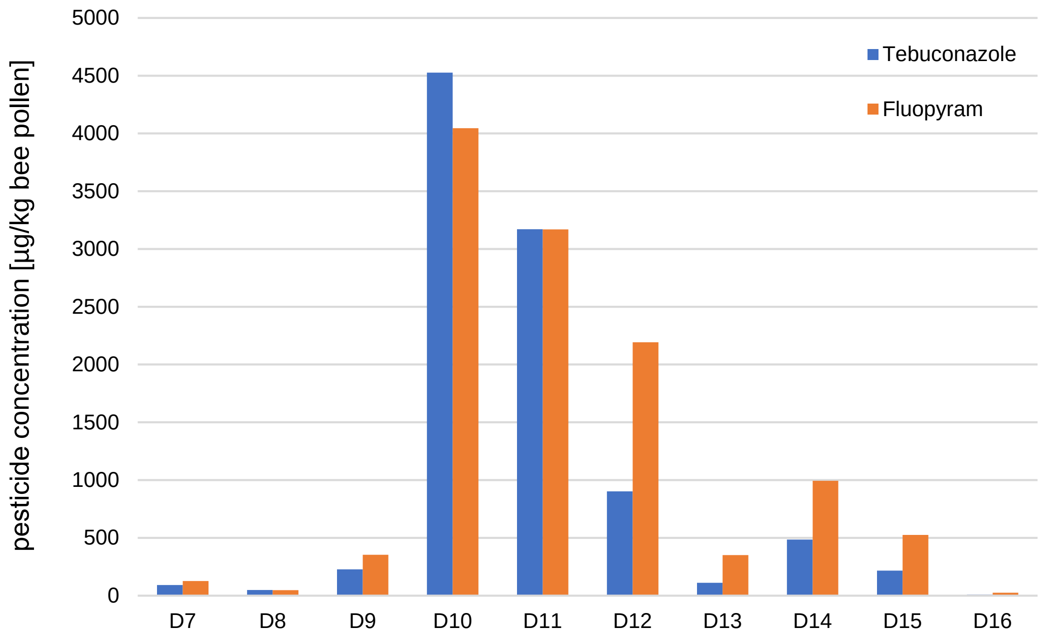

Fig. S4 Day-to-day progression of all pesticides with maximum concentrations < 100 ng/g bee pollen

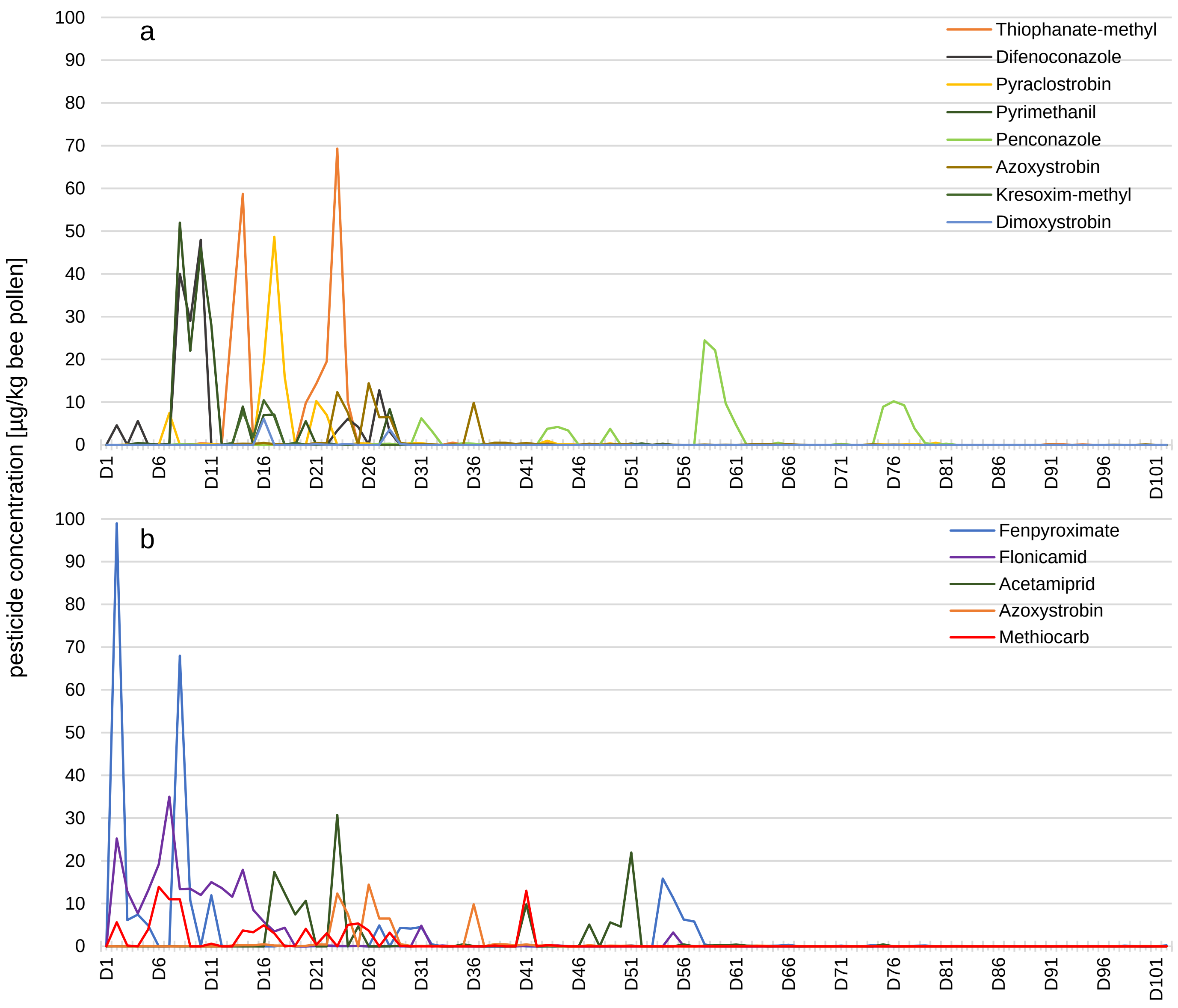

Fig. S5 Hierarchical Clustering of 13 pesticides with maximum concentrations of < 100 ng/g bee pollen

Hierarchical Clustering was performed by using the computer software JMP® pro 15.0 (SAS Institute Inc, Cary, NC, USA)

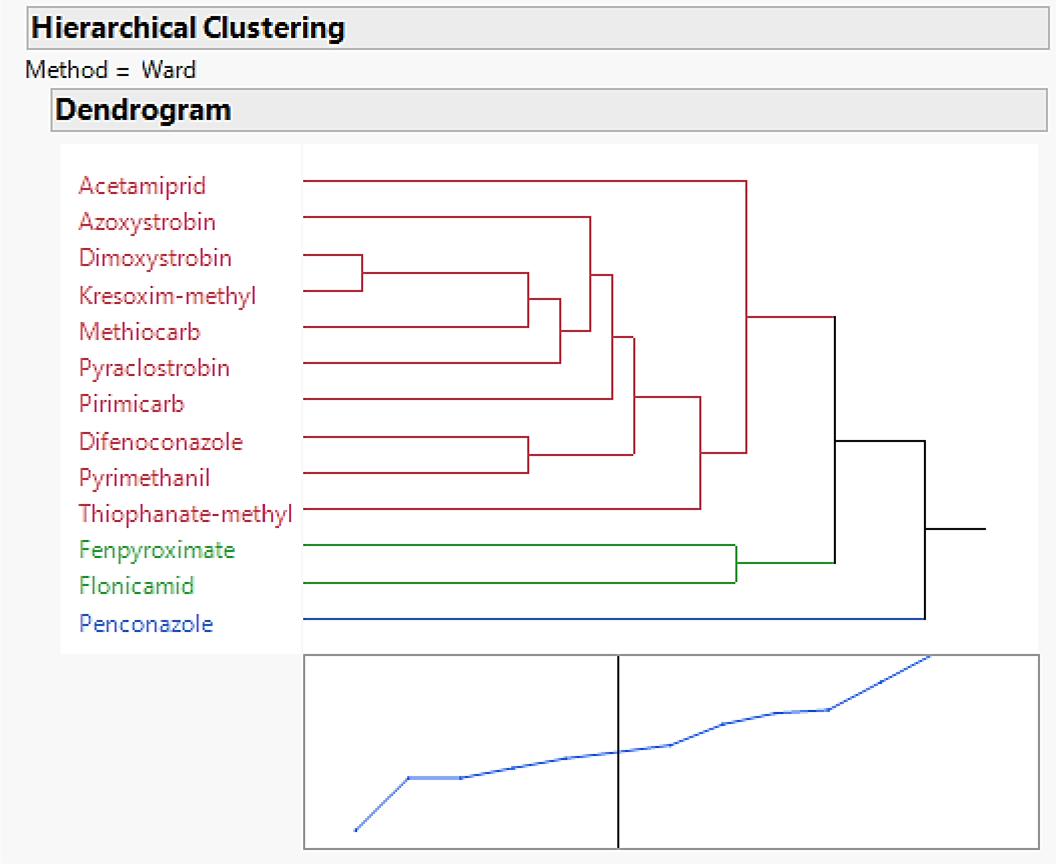

Supplement: Supplementary file 2 — (PDF 467 kb) [file 11356_2020_12318_MOESM2_ESM.pdf]
